# Using the Hephaistos orthotic device to study countermeasure effectiveness of neuromuscular electrical stimulation and dietary lupin protein supplementation, a randomised controlled trial

**DOI:** 10.1371/journal.pone.0171562

**Published:** 2017-02-16

**Authors:** Jochen Zange, Kathrin Schopen, Kirsten Albracht, Darius A. Gerlach, Petra Frings-Meuthen, Nicola A. Maffiuletti, Wilhelm Bloch, Jörn Rittweger

**Affiliations:** 1 Division of Space Physiology, Institute of Aerospace Medicine, German Aerospace Center (DLR), Cologne, Germany; 2 Department of Molecular and Cellular Sport Medicine, German Sport University Cologne, Cologne, Germany; 3 Institute of Biomechanics and Orthopaedics, German Sport University Cologne, Cologne, Germany; 4 Human Performance Lab, Schulthess Clinic, Zürich, Switzerland; 5 Department of Paediatrics and Adolescent Medicine, University of Cologne, Cologne, Germany; Universite de Nantes, FRANCE

## Abstract

**Purpose:**

The present study investigated whether neuromuscular electrical stimulation for 20 min twice a day with an electrode placed over the soleus muscle and nutritional supplementation with 19 g of protein rich lupin seeds can reduce the loss in volume and strength of the human calf musculature during long term unloading by wearing an orthotic unloading device.

**Methods:**

Thirteen healthy male subjects (age of 26.4 ± 3.7 years) wore a Hephaistos orthosis one leg for 60 days during all habitual activities. The leg side was randomly chosen for every subject. Six subjects only wore the orthosis as control group, and 7 subjects additionally received the countermeasure consisting of neuromuscular electrical stimulation of the soleus and lateral gastrocnemius muscles and lupin protein supplementation. Twenty-eight days before and on the penultimate day of the intervention cross-sectional images of the calf muscles were taken by magnetic resonance imaging (controls n = 5), and maximum voluntary torque (controls n = 6) of foot plantar flexion was estimated under isometric (extended knee, 90° knee flexion) and isokinetic conditions (extended knee), respectively.

**Results:**

After 58 days of wearing the orthosis the percentage loss of volume in the entire triceps surae muscle of the control subjects (-11.9 ± 4.4%, mean ± standard deviation) was reduced by the countermeasure (-3.5 ± 7.2%, p = 0.032). Wearing the orthosis generally reduced plantar flexion torques values, however, only when testing isometric contraction at 90° knee ankle the countermeasure effected a significantly lower percentage decrease of torque (-9.7 ± 7.2%, mean ± SD) in comparison with controls (-22.3 ± 11.2%, p = 0.032).

**Conclusion:**

Unloading of calf musculature by an orthotic device resulted in the expected loss of muscle volume and maximum of plantar flexion torque. Neuromuscular electrical muscle stimulation and lupin protein supplementation could significantly reduce the process of atrophy.

**Trial registration:**

ClinicalTrials.gov, identifier NCT02698878

## Introduction

Chronic unloading of skeletal muscles commonly results in muscle atrophy, which is more pronounced in muscles involved in locomotion than in muscles predominantly involved in joint stabilization [1]. In patients, muscle atrophy is a negative side effect of immobilization by bed rest or by orthopaedic devices which are used to protect healing tissues. Astronauts represent a special group of healthy persons who are severely affected by atrophy of leg and back musculature because of microgravity-induced chronic unloading. During long term space mission on board the former Soviet/Russian space station Mir or on the current International Space Station ISS exercise countermeasures have been obligatory. However, formerly used countermeasures have only been partially effective in preserving leg and back muscle volume and function [2–5], whereas in recent years astronauts who trained on the novel Advanced Resistive Exercise Device (ARED) returned back from space in a visibly better condition [6](personal observations). However, ARED training is time-consuming and connected with the potential risk of injury by high loads. Therefore the development of more efficient and safer for countermeasures conserving muscles and bones in space is still an ongoing challenge.

Countermeasures against microgravity effects on the human body are typically developed and verified using the ground based models of long term bed rest [7–9], or unilateral limb suspension [10] for inducing muscle atrophy. However, the bed rest model cannot distinguish local and systemic immobilization effects, which to a lesser extent also applies for unilateral limb suspension. Therefore, we recently developed the novel Hephaistos orthosis [11,12], which provides a selective unloading model for the calf musculature. Subjects wearing the Hephaistos orthosis can walk more or less normally [11] without crutches and without immobilization of knee and ankle, respectively, which are both obligatory in full casts as well as in the unilateral limb suspension model. The Hephaistos orthosis significantly reduces the activity of calf muscles during walking [11]. After 56 days of wearing the orthosis maximum isokinetic plantar flexion torque was reduced by about 23% and soleus muscle fibre cross-section was reduced by about 9% [13]. The present study is the first one to use the Hephaistos orthosis for testing countermeasures.

It is well-established that neuromuscular electrical stimulation (NMES) can counteract the atrophy of immobilised skeletal muscles. This was shown e.g. in experimental studies on mammalian animals [14–17] as well as in clinical studies on patients immobilised by long term bed rest due to general illness [18,19], or by orthopaedic [20–24] or neurological [25] conditions. NMES activates signalling pathways controlling the synthesis of proteins involved in growth and differentiation of muscle fibres, in muscle energy metabolism, in the activation of satellite cells, etc. [20,26,27].

The growth stimulus provided by muscular training, including by means of NMES, is frequently supported by nutritional protein supplementation in order to optimise the metabolic conditions for muscle protein synthesis in terms of the availability of the different amino-acids [28–35]. However, in most cases this protein is of animal origin which is rich in sulphur-containing amino acids causing a moderate metabolic acid load. A metabolic acidification would stimulate bone resorption and muscle protein degradation [36–38]. To reduce the risk of a potentially occurring acidosis and its negative effects on bone and muscle, which at least would partially counteract the benefits of supplementation with animal protein, in this study lupin seeds were applied as nutritional supplementation. The vegetable seeds contain up to 40% of protein and due to a lower amount of sulphur-containing amino acids (lupin flour analysis: methionine: 0.13 g / 100 g; cysteine: 0.61g/100g) than whey protein for example (methionine: 2.28g/100g; cysteine: 2.41g/100g) result in a lower metabolic acidification than animal protein.

The NutriHEP study (ClinicalTrials.gov identifier NCT02698878) focussed on the local and the systemic alteration in glucose metabolism in the soleus muscle during atrophy induced by partial unloading with the Hephaistos orthosis and the effectiveness of NMES plus lupin protein supplementation for reducing muscle atrophy induced by the orthosis and the effects of atrophy on glucose metabolism. Out of the greater NutriHEP study, this first paper describes the study design and the principal structural and functional alterations induced by the orthosis, which are the losses in calf muscle volume and maximal voluntary strength, as well as the effectiveness of the applied countermeasures for minimizing the expected loss in muscle volume and strength.

## Methods

### Subjects

**Fig 1:**
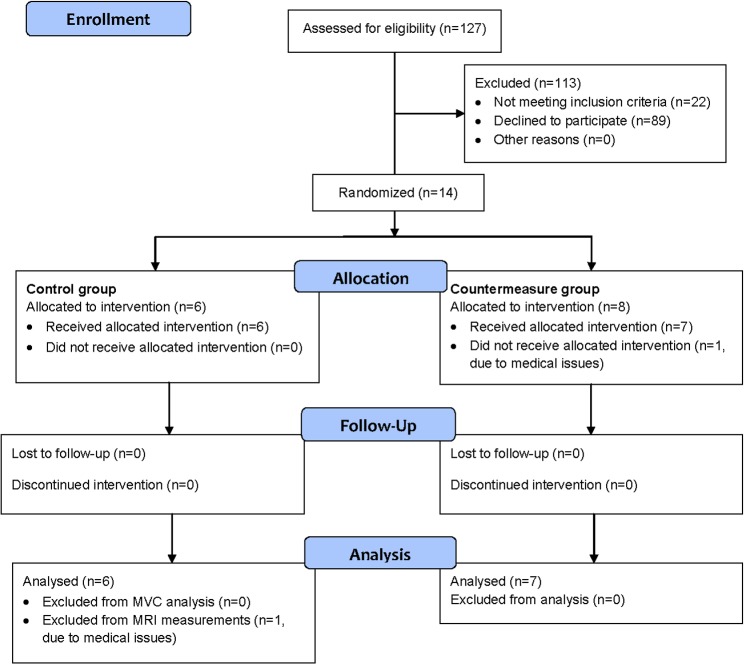
CONSORT 2010 Flow Diagram. The diagram shows the recruitment, allocation and analysis of subjects in the NutriHEP-study with respect to the orthosis intervention and the examinations concerning maximum voluntary contraction (MVC) and magnetic resonance imaging (MRI)

Thirteen healthy male subjects (Fig 1) with an age of 26.4 ± 3.7 years (mean ± standard deviation (SD)) and a body mass index of 22.9 ±1.6 kg/m^2^ completed the intervention phase and the maximal voluntary strength test. Only 12 out of 13 subjects (age of 27.3 ±3.9 years, body mass index of 23.0 ±1.54 kg/m^2^) participated in the MRI experiments (Fig 1). Subjects were enrolled between April 1^st^ and August 3^rd^ 2014. All measurements were performed at the German Aerospace Center, DLR, in Cologne, Germany.

The entire NutriHEP-study comprising all experiments including those reported here was registered on 23^rd^ February 2016 at ClinicalTrials.gov identifier NCT02698878 in response to the altered policy of PLOS-ONE assigning physiological studies on healthy subjects as clinical trials that must be registered. The NutriHEP study complied with the Declaration of Helsinki and was approved on the 25^th^ April 2014 by the ethics committee of the Ärztekammer Nordrhein, Düsseldorf, Germany.All subjects gave their written informed consent before study inclusion.

### Study design

For 60 days, all subjects wore the Hephaistos orthosis during their regular life activity. Whether a subject wore the orthotic device on the left or the right leg was assigned randomly by tossing a coin. Each orthosis was individually adapted by ORTEMA GmbH (Markgröningen, Germany). A temperature probe (Orthotimer, rollerwerk medical engineering & consulting, Balingen, Germany) integrated in the orthosis recorded the daily duration of wearing the orthosis.

Six subjects were only wearing the orthosis (control group). Seven subjects additionally underwent the countermeasure including daily lupin protein supplementation and NMES twice a day. Groups of two subjects were allocated to the two study groups by tossing a coin. One subject of the control was excluded from MRI examination (Fig 1).

### Countermeasures

NMES was applied to the soleus muscle two times per day using the T-ONE MEDIPRO device (I.A.C.E.R. srl, Martellago, Italy). A 5 × 5 cm stimulation electrode was positioned on the soleus muscle lateral and distal from the lower edge of the gastrocnemius lateralis muscle. A further accessory stimulation electrode was placed over the belly of the gastrocnemius lateralis muscle, where the electrical current likely reached both, the thin lateral gastrocnemius muscle and the underlying proximal part of the soleus muscle. The reference electrode (5 × 10 cm) was placed transversally on the proximal aspect of the two gastrocnemii. The stimulator delivered pre-programmed biphasic rectangular pulses with the following characteristics: frequency of 30 Hz, pulse duration of 200 μs, on/off ratio of 5/5 s with a ramp-up of 1 s and a ramp-down of 0.5 s. Subjects were asked to constantly increase current intensity during each session to the maximally tolerated level (range: 0–100 mA), while they were seated with the knee flexed at approximately 90°. This knee angle minimizes the contribution of gastrocnemius muscles to plantar flexion [39]. Subjects were asked to minimize heel rising by the contraction of the stimulated plantar flexor muscles by holding down the knee of the stimulated leg with both hands. The total duration of a treatment session was 20 min (40 min/day).

Every day for the whole duration of the intervention subjects of the countermeasure group received 19g of lupin crunchy which they mixed with their regular food. The amount of crunchy was independent of body weight and based on the literature [40,41] considering a conglutin gamma content of 4% of the total protein and a protein content of 41.3% in the lupin crunchy (Eurofins Laborservice GmbH, Cologne, Germany). The content of sulphur containing amino acids methionine and cysteine for whey and lupin protein (analysed as lupin flour) and the content of protein in lupin crunchy were analysed by a local laboratory (Eurofins Laborservice GmbH, Cologne, Germany).

### Assessment of muscle volume using MRI

MRI examinations of the calf musculature were conducted 28 days before and on the 58^th^ day of wearing the orthosis. Both MRI measurements were scheduled on the day before the biopsies from the soleus muscle were taken. The first biopsy was taken almost four weeks before wearing the orthosis to reduce the risk of thrombosis. MRI acquisitions were obtained from a 3T scanner (mMR Biograph (PET-MRI scanner based on the Verio system), Siemens, Erlangen, Germany) with the the Verio’s standard body coil. During the examination the lower leg was placed in a mono-resonant sodium birdcage coil (Rapid Biomedical, Würzburg, Germany) for another experiment. Changes in muscle volume were determined from ^1^H-MR images recorded with the body coil.

All examinations were carried out at rest and in the supine position. Prior to MRI scanning, subjects were resting for 30 min in the supine position to reach a constant distribution of the interstitial volume before the measurements started. Subjects were then transferred into the scanner room and positioned for examination. The belly of the calf musculature from the orthosis leg was positioned in volume of homogenous sensitive of the sodium coil. To avoid motion and to achieve a defined and reproducible region of the calf musculature for pre and post measurements, the leg was supported by cushions and stabilized with a wooden footrest. The distance between the footrest and the sodium coil was individually defined for each subject. The first muscle volume measurement started after 45 min of supine rest after the sodium MRI experiment.

For the assessment of muscle volume a series of trans-axial 2-dimensional PD-weighted Dixon TSE (turbo spin echo) sequence was used with the following parameters: acquisition time (TA) 2:48 min, pulse repetition time (TR) 3000 ms, echo time (TE) 11 ms, flip angle 180°., bandwidth 292 Hz/Px. The field of view covered a distance of 16.5 cm with series of 17 images. Each image had an area of 192 x 192 mm with a 256 x 256 pixel matrix resulting in a resolution of 0.75 mm x 0.75 mm per pixel. The slice thickness was 5 mm with a gap of 5 mm in-between slices.

In each image the individual areas of the gastrocnemius lateralis muscle, the gastrocnemius medialis muscle, and the soleus muscle were segmented using custom made software (ROI-segmenter, University of Applied Technology Niederrhein, Institute for Pattern Recognition, Krefeld, Germany). Before and after the intervention, for all series of images covering identical regions of the lower legs the sum areas were calculated and multiplied by slice thickness (5 mm). For each examination the volume of the whole triceps surae muscle was calculated as the sum of its three heads. The percent changes in muscle volumes pre and post intervention were calculated as 100 x (pre–post)/pre.

### Assessment of maximum voluntary strength

Maximum voluntary strength of the plantar flexors was evaluated in three different modes: 1) isometric, with the knee almost extended and a neutral foot position (internal angle: 90°), 2) isokinetic concentric, with the knee almost extended and an angular velocity of 60°/s, and 3) isometric with hip and knee joints flexed at 90° and ankle joint in 10° of dorsiflexion (internal angle: 80°).

Maximum voluntary strength in modes 1 and 2 was tested using the Isomed 2000 dynamometer (D&R Ferstl GmbH, Hemau, Germany), which measures instantaneous torque in Nm. Subject laid supine with their knees almost extended and the foot of the orthosis wearing leg fixated on a pedal attached to the dynamometer. For these two tests on the dynamometer subjects completed a warm-up of 3 sets of 5 consecutive submaximal isokinetic plantar- and dorsal flexions at 60°/s. The warm-up was followed by 2 sets of 5 consecutive isokinetic, concentric-concentric plantar- and dorsal flexions at 60°/s with only plantar flexion performed at maximal intensity. Subsequently, maximum isometric plantar flexion torque was evaluated during 3 contractions of 3 to 5 s duration. Rest periods of 1 min were consistently utilized between sets (isokinetic mode) and contractions (isometric mode).

Maximum isometric plantar flexion torque was also evaluated with the knee flexed at 90° (mode 3) by means of a custom made device. The subject was seated on a chair and the foot of the tested leg was placed in 10° dorsiflexion on a footplate, which was adjusted such that the rotational axis of the plate corresponded to that of the ankle joint. A strain gauge sensor was placed under the footplate with a fixed lever to assess the torques around the rotational axis of the device Thighs were prevented from moving up during maximal plantar flexion efforts using a bended and padded metal plate placed above the knee. To adjust the device according to the individual lengths of lower legs, the metal plate could be height adjusted by a threaded rod within a strong metal frame. After a brief warm-up consisting of 15 to 20 repetitive sub-maximal isometric plantar flexions, subjects performed two maximal isometric plantarflexion efforts of 3 to 5 s duration with a 2-min rest period. In case the differences in maximum torque between the two trials was higher than 5% an additional trial was performed.

Only the highest torque value reached during the different maximal contractions was retained for all three testing modes.

### Statistical analyses

IBM SPSS statistics software (version 21) was used for data evaluation. Absolute volume and torque values given in ml or Nm, respectively, were tested with the fixed factors phase (pre, post) and group (control, countermeasure) as well as phase*group using linear mixed models (LME) for repeated measurements (phase per subject, covariance structure was set to “diagonal”). Where the repeated measure LME did reach convergence for absolute values, we also applied LME using phase as a simple fixed factor. Delta-values of volume and torque (pre-post) were calculated and given in ml and Nm, respectively, or in %. Delta-values in % were tested with LME using only the fixed factor group. Furthermore, for each group pre- and post- values of volume and torque were tested for normality using the Kolmogorov-Smirnov-test. Subsequently, pre- and post-values were group wise tested for significant differences using paired t-test. A significance level of p<0.05 was defined. An effect size of 1.52 was calculated using the R-project package “basic functions of power analysis” with the function “power calculations for two samples (different sizes) t-tests of mean” and the parameters n1 = 6 and n2 = 7 as numbers of observation, significance level = 0.05, power = 0.7, and the two sided hypothesis alternative. Expecting a relative loss in isokinetic torque of -23.4% ± 8.2% in the control group [13] the minimum significant countermeasure effect would reduce the loss in maximum isokinetic torque to about -10.9%.

## Results

The control group wore the orthosis for 12.6 h/day (±2.0 (SD), minimum 10.2 h/day, maximum 15.9 h/day). The countermeasure group wore the orthosis for 11.4 h/day (±1.5 (SD), 8.3 to 12.8 h/day, p = 0.358).

**Fig 2:**
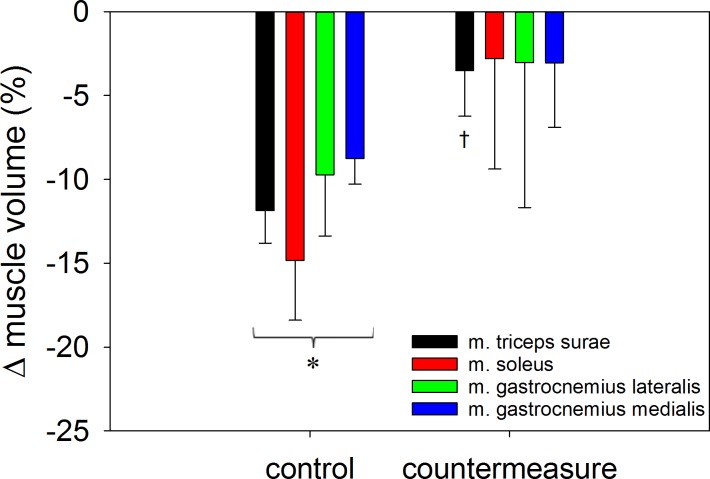
Percent changes (means ± standard error of the mean) in the triceps surae muscle and its three parts by the orthosis intervention in the control and in the countermeasure group, respectively. *: p<0.05 for the delta-value versus 0. †: p<0.05 for differences between the two groups.

In subjects from the control group the volume of the triceps surae muscle was reduced by -11.9 ± 4.4% (mean ± SD). Volume loss was significantly less in the countermeasure group (-3.5 ± 7.2%) than in the control group (p = 0.044, Fig 2, Table 1). In control subjects, volume was significantly reduced in all three heads of the triceps surae muscle. As a trend the greatest effect was observed for the soleus muscle. In the countermeasure group, volume losses of the individual heads of triceps surae muscle were not significantly different from 0. However, the countermeasure effect also did not reach statistical significance when comparing the two subject groups for the single parts of the triceps surae muscle (Fig 2, Tables 1 and 2).

**Table 1:** Significance (p-values) of the percent decreases in muscle volume and strength induced by wearing the orthosis with and without the countermeasures tested by LME and t-tests (compare Figs 2 and 3).

	LME, constant term vs. 0	LME, intervention	paired t-test countermeasure group	paired t-test control group
**muscle volume**				
gastrocnemius medialis	**0.033**	0.261	0.458	**0.005**
gastrocnemius lateralis	0.266	0.549	0.739	0.056
soleus	0.063	0.184	0.684	**0.014**
triceps surae	**0.002**	0.044	0.242	**0.004**
**muscle strength**				
isometric, knee extended	**0.010**	0.765	**0.035**	0.115
isometric, knee flexed	**<0.001**	**0.032**	**0.012**	**0.005**
isokinetic, knee extended	**0.017**	0.801	**0.013**	0.242

**Table 2:** Muscle volume (means ± standard deviation, ml) of the triceps surae muscle and its three heads

muscle volume (ml)	intervention	pre	post	post—pre
gastrocnemius medialis	countermeasure	116.6± 29.5	112.1 ± 26.7	-4.4± 12.1
	control	115.4 ± 37.5	104.6 ± 30.3	-10.8 ± 8.2 †
gastrocnemius lateralis	countermeasure	55.3 ± 23.4	53.2 ± 24.5	-2.1 ± 11.8
	control	66.0 ± 19.7	59.4 ± 16.9	-6.5 ± 6.7
soleus *	countermeasure	214.6 ± 31.0	205.2 ± 25.2	-9.4 ± 31.4
	control	200.3 ± 27.0	170.8 ± 27.1	-29.6 ± 15.4 †
triceps surae	countermeasure	386.5 ± 67.3	370.5 ± 54.1	-16.0 ± 23.8
	control	381.7 ± 71.6	334.7 ± 50.1	-47.0 ± 26.0 †

Muscle volume was measured in 17 slices of 5 mm thickness located around the belly of the calf with 5 mm gaps in between the slices. Measurements were made before and after 58 days of wearing the orthosis.

Countermeasure effect: * p<0.05, LME.

Delta-Value different from 0: † p<0.05, t-test.

**Fig 3:**
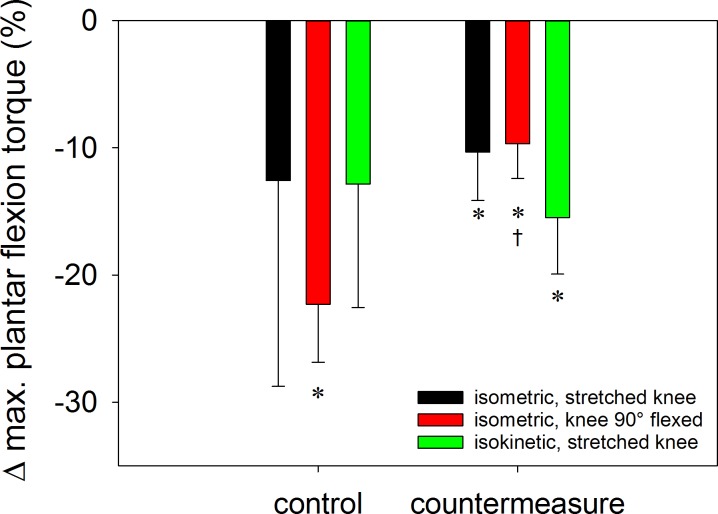
Percent changes (means ± standard error of the mean) in maximal voluntary plantar flexion torque by the orthosis intervention in the control and in the countermeasure group, respectively. *: p<0.05 for the delta-value versus 0. †: p<0.05 for differences between the two groups.

Significant effects on both, wearing of the orthosis and the combination of countermeasures on calf muscle strength was only found for the isometric test with the knee flexed at 90°. Under these conditions maximal voluntary torque of the control group decreased by -22.3 ± 11.2% (mean ±SD) versus -9.7 ± 7.2% for the countermeasure group (p = 0.032, Fig 3, Tables 1 and 3). The isometric and isokinetic tests performed with the knee extended showed a loss of strength for both groups (constant term by LME, Tables 1 and 3) with no difference between the control and the countermeasure subjects (Table 1). When both groups were analysed separately at post-intervention, only the countermeasure group showed a significant decrease in isometric and isokinetic torque with the knee extended (Table 1, Fig 3).

**Table 3:** Maximum voluntary contraction torque (means ± standard deviation, Nm) of the plantar flexors in three different modes

torque (Nm)	intervention	pre	post	post-pre
isometric, extended knee	countermeasure	244 ± 42	215 ± 22	-28 ± 28 †
	control	221 ± 50	195 ± 59	-26± 29
isometric, knee flexed *	countermeasure	220 ± 38	198 ± 30	-22 ± 18 †
	control	242 ± 46	186 ± 37	-56 ± 32 †
isokinetic, extended knee *, #	countermeasure	155 ± 28	129 ± 8	-27 ± 22 †
	control	129 ± 16	112 ± 31	-22± 25

Measurements were made before and after 58 days of wearing the orthosis.

Orthosis effect: * p<0.05, LME.

Countermeasure effect: # p<0.05, LME.

Delta-Value different from 0: † p<0.05, t-test.

## Discussion

In this ambulatory study the Hephaistos orthosis subjects wore the Hephaistos orthosis between 8 and 16 h/day which indicated a good tolerance of the orthosis and high compliance of the subjects during their daily walking activity. After two short familiarization sessions before the intervention phase, subjects accustomed quickly to the usage of the orthosis. Only one subject reported excessive pressure caused by the upper edge of the shin pad of the orthosis, but this problem was resolved by the manufacturer during the first week of unloading.

Wearing the Hephaistos orthosis for 60 days resulted in the expected decrease in triceps surae muscle volume and plantar flexion strength. Both MRI and maximum voluntary torque results at 90° of knee flexion demonstrated that volume and strength loss were greater in the mono-articular soleus muscle than in the bi-articular gastrocnemii. NMES together with the nutritional supplementation of protein rich lupin seeds resulted in significantly lower decreases in triceps surae muscle volume and plantar flexion strength compared to the control group. These results are remarkable, because the benefits in this study were obtained with comparably little effort for the subjects (40 min/day of NMES and easy-to-control nutritional supplementation). So far, preventing muscle atrophy during bed rest was only fully effective when countermeasures such as resistance training were performed at least on a daily basis and with very forceful contractions [42,43].

Even if the intensity of the contractions evoked by NMES was not controlled in this study, due to practical reasons (all NMES sessions were conducted at home and not in a laboratory), we expect that–based on average current intensities and settings comparable to previous studies [24,44,45] we conducted–plantar flexion force evoked by NMES was close to 50% of pre-intervention maximal voluntary strength.

NMES therapy has been shown to be effective in treating skeletal muscles–the quadriceps in particular–during prolonged periods of disuse/immobilization due to injury, surgery or illness, as it has the potential to preserve muscle protein synthesis and prevent muscle atrophy [24]. Probably due to its peculiar motor unit recruitment pattern (superficial, incomplete, asynchronous and non-selective [46]), NMES has also been shown to enhance whole-body glucose uptake in healthy subjects [47] and to maintain glycemic control in critically-ill patients [48] by preventing muscle-specific AMPK failure, restoring GLUT4 disposition, and diminishing protein breakdown. Interestingly, a short period of NMES also prevented type 2 muscle atrophy in this latter study [48], which represents an important finding for the treatment of the typical disuse atrophy. In the current study, the NMES treatment was quite unique because of (1) the unloading model, (2) the combined implementation with protein supplementation and (3) the application of NMES exclusively to the triceps surae muscle of one side, which was quite a minor stimulus for preserving muscle protein synthesis as well as for countering neuromuscular and functional alterations induced by disuse. Nevertheless, our muscle volume and strength results are quite solid, despite the small sample size, and confirm the effectiveness of NMES as a countermeasure to disuse-induced muscle atrophy and weakness for a muscle group other than the quadriceps.

However, and despite the fact that wearing the Hephaistos orthosis leads to a muscle weakening that is comparable to bed rest, there is also a possibility that countermeasures could generally be more effective with the Hephaistos than during bed rest.

As with all studies, there are some limitations that need to be considered. Firstly, this was a comparatively small study, and therefore caution must be taken when generalizing the findings. Secondly, it remains unclear from this study what the contribution from the two constituents of the countermeasure was. However, there is a genuine possibility that the combination of protein supplementation and NMES could be more effective than each of the two on its own. Therefore, we feel that the study rationale of combining the two countermeasures in the first approach was justified and that one should now go ahead and disentangle the partial contribution of each of the two countermeasures.

In conclusion, the present study has successfully used the novel Hephaistos unloading device to study the effectiveness of a combined lupin protein / NMES countermeasure against calf muscle atrophy. The countermeasure showed good effectiveness to preserve calf muscle volume, and to a somewhat lesser extent also to preserve plantar flexor strength. Results are encouraging to further explore this combined countermeasure. Moreover, we feel the unloading model also lends itself to study immobilization effects and countermeasure efficacy in populations that cannot be easily be studied by bed rest, such as older people.

## Supporting information

S1 File Ethics application to the Ärztekammer Nordrhein. This file includes also all information about the protocol of the entire study.
(PDF)

S2 File Zip-File containing SPSS-data files listing the individual MRI and torque data of all subjects. (ZIP)

S3 File PONE-D-16-27158-CONSORT 2010 Checklist. (DOC)
