# Supplementary material for: Using the Hephaistos orthotic device to study countermeasure effectiveness of neuromuscular electrical stimulation and dietary lupin protein supplementation, a randomised controlled trial
Source: PLoS One. 2017 Feb 16;12(2):e0171562. doi: 10.1371/journal.pone.0171562 (PMC5313207; doi:10.1371/journal.pone.0171562)
Supplement: S1 File — This file includes also all information about the protocol of the entire study. (PDF) [file pone.0171562.s001.pdf]

Institut für Luft- und
Raumfahrtmedizin

Ethics application to the Ärztekammer Nordrhein for the study:

Investigating the Influence of NUTRItion and Neuromuscular Stimulation on Local Insulin Sensitivity in *triceps surae muscle* during Immobilization using the HEPhaistos Orthosis (NUTRIHEP)

Contents

Formal 4
1.1 Name of the Project 4
1.2 Applicant and Executive Medical Director.... 4
1.3 Measurements 4
1.4 Participating Physicians/Scientists.... 4
1.5 Cost Object/Sponsor.... 6
2 Test Plan 7
2.1 Name of the Project 7
2.1.1 The Objectives of the Proposed Study 9
2.1.2 The Hephaistos Orthosis 9
2.1.3 Lupine Seeds.... 10
2.1.4 Neuromuscular Electrical Stimulation (NMES) 11
2.1.5 Anodal transcranial direct current stimulation (tDCS) 11
2.2 Study Medication / Dosage Form....12
2.3 Baseline Data Collection Pharmacological-Toxicological Testing....12
2.4 Type of the Study....12
2.5 Nutritional Pre-Study to test the Acute Effect of Lupine Flour in Comparison to Whey Protein 12
2.6 Study Implementation 16
2.7 The Introduction and Explanation to the Subjects 20
2.8 The Methods Adopted in the Proposed Study 21
3 The following legislation is applicable....41
4 The Intended duration of Proposed Study 42
5 Subjects Selection 42
5.1 Inclusion Criteria for the Pre-Study....42
5.2 Exclusion Criteria for the Pre-Study....42
5.3 Inclusion Criteria for the Main Study....43
5.4 Exclusion Criteria for the Main Study....43
5.5 Additional Exclusion Criteria for MRI 44
5.6 Statistical Analysis....45

5.7 Number of Subjects 45
5.8 Inclusion of subjects under the age of 50 (§28d Abs. 3 RöV) 45
5.9 Criteria for Randomizations 46
5.10 Drop-out Reasons:...46
6 Potential Adverse Effects..47
6.1 Complications and risk resulting from methods 47
6.1.1 Neuromuscular Electrical Stimulation (NMES) 47
6.1.2 Lupine Seeds.. 47
6.1.3 Muscle Biopsies.. 47
6.1.4 Microdialysis 48
6.1.5 Hyperinsulinemic Euglycemic Clamp.. 48
6.1.6 Basal Metabolic Rate.. 48
6.1.7 Bone densitometry.. 49
6.1.8 Maximum Voluntary Contractions (MVC).. 49
6.1.9 Magnetic Resonance Imaging (MRI) 49
6.1.10 Vertical Jumping Test 50
6.1.11 Blood 50
6.1.12 The Hephaistos Orhtosis 50
6.1.13 Ergometric device / Quick release 50
6.1.14 Passive Range of Motion.. 51
6.1.15 Muscle Architecture at Rest.. 51
6.1.16 Peripheral Nerve Stimulation (PNS) 51
6.1.17 Activation Capacity (AC).. 52
6.1.18 Transkranielle Magnetstimulation 52
6.1.19 Tendon and Aponeurosis Mechanical Properties 52
7 Risk-Benefit Analysis..52
8 Insurance Protection...53
9 Signature 55
10 Appendix..57
11 References 58

Formal

1.1 Name of the Project

Investigating the Influence of NUTRItion and Neuromuscular Stimulation on Local Insulin Sensitivity in *triceps surae muscle* during Immobilization using the HEPhaistos Orthosis (NUTRIHEP)

1.2 Applicant and Executive Medical Director

Prof. Dr. Jörn Rittweger
Institute of Aerospace Medicine, DLR
Linder Höhe
D-51147 Cologne
Tel.: 2203 601 3080
Fax: 2203 611 59

1.3 Measurements

1.4 Participating Physicians/Scientists

Prof. Dr. Jörn Rittweger
Dr. Petra Frings-Meuthen
PD Dr. Jochen Zange
Dr. Bergita Ganse
Kathrin Schopen
Darius Kornetka
Ann Charlotte Ewald
Institute of Aerospace Medicine, DLR Cologne
Linder Höhe

D-51147 Cologne
Tel.: +49 2203-601- 3360
Fax: +49 2203-61159

Prof. Dr. Med. Wilhelm Bloch
Department of Molecular and Cellular Sport Medicine, German Sport University, Cologne
Am Sportpark Müngersdorf 6
50933 Cologne
Tel.: +49 221 4982-5380
Fax: +49 221 4982-8370

Prof. Dr. Albert Gollhofer
Dr. Christian Leukel
Albert-Ludwigs-University Freiburg
Institute for Sports and Sports Sciences
Schwarzwaldstraße 175
79117 Freiburg
Tel.: +49 761 203 – 4510
Fax: +49 761 203 – 4534

Dr. Michael Boschmann
Charite Universitätsmedizin Berlin
Campus Berlin-Buch
Franz-Volhard-Centrum für Klinische Forschung am
Experimental & Clinical Research Center (ECRC)
Lindenberger Weg 80
13125 Berlin
Deutschland
Telefon: +49(0)30 450 540 241
Telefax: +49(0)30 450 540 920

Nicola A. Maffiuletti, PhD
Head, Neuromuscular Research Lab

Schulthess Clinic
Lengghalde 2 - 8008 Zurich
Switzerland
Phone +41 44 385 7579

Dr. Kirsten Albracht
Institute of Biomechanics and Orthopaedics
German Sport University Cologne
Am Sportpark Müngersdorf 6
50933 Köln
Tel.: +49 (0)221 4982-5680
Fax: +49 (0)221 4971598

Jean-François Grosset, PhD
Université de Technologie de Compiègne (UTC)
UMR CNRS 7338 Biomécanique et Bioingénierie
BP20529
60205 Compiègne Cedex, France
tel : 33 (0)3 44 23 43 92

1.5 Cost Object/Sponsor

DLR- Institute of Aerospace Medicine
Department: Space Physiology
Cost object: 2 475 101
Cost unit: 31612
Linder Höhe, D-51147 Cologne

2 Test Plan

2.1 Name of the Project

Investigating the Influence of NUTRItion and Neuromuscular Stimulation on Local Insulin Sensitivity in *triceps surae muscle* during Immobilization using the HEPhaistos Orthosis

Long-term immobilization was shown to cause insulin resistance in healthy men. During space flight astronauts are exposed to weightlessness which does have a similar effect on the human body as immobilization. Modeling weightlessness by head-down bed rest, Blanc et al. (2000) demonstrated increased plasma levels of insulin during seven days of bed rest in both men and women while plasma glucose levels remained unchanged in men but were slightly decreased in women. Accordingly, higher insulin to glucose ratio could be observed in both cases suggesting the occurrence of a slight insulin resistance (Blanc et al, 2000). Supporting this hypothesis Alibegovic et al. (2009) report that nine days of bed rest induced whole body insulin resistance in both healthy men and first-degree relatives of patients with Type 2 diabetes (Alibegovic et al., 2009). Furthermore, Stein et al observed an increase in insulin production in 17 days of bed rest and insulin resistance during space flight (Stein et al., 1997, Stein et al., 1994). Impairment of insulin sensitivity and glucose metabolism seem to be linked to a loss of muscle mass which occurs due to immobilization and disuse of the muscle. Because gravitational forces are lacking in microgravity, muscles become mechanically unloaded during spaceflight resulting in the loss of muscle mass and insulin insensitivity.

To get further insights into the molecular mechanism associated with the development of insulin resistance Weber-Carstens et al. (2013) investigated the translocation of the glucose transporter type 4 (GLUT4) in skeletal muscle in critically ill patients. Whilst GLUT4 was trapped at perinuclear spaces in critically ill patients, GLUT4 was repositioned to the membrane in these patients by electrical stimulation of the muscle (Weber-Carstens et al., 2013).

The translocation of GLUT4 is known to occur due to two different signaling pathways within the cell. On the one hand translocation is induced by contraction of

the muscle while the other pathway is insulin-dependent. To further investigate how insulin sensitivity can be improved during immobilization we want to immobilize and unload the soleus and gastrocnemius muscle in the lower leg of healthy male subjects by using a special orthosis – The Hephaistos orthosis. In a previous study implemented at the German Aerospace Center in Cologne (HEP-Study) it was shown that wearing this orthosis on one leg for 56 days engendered a sizeable unloading response of both the soleus and the gastrocnemius muscles. We now wish to elucidate whether the local insulin sensitivity can be improved within the unloading and atrophy response during 60 days. Moreover, it is intended to explore whether insulin sensitivity can be improved by addressing both the insulin- and the contraction-dependent signaling pathway of GLUT4 translocation. To improve insulin-dependent glucose uptake, subjects will include lupine seeds. A rationale for this is provided by, Bertoglio et al. who demonstrated glucose lowering effects of lupin protein in healthy subjects in 2011 (Bertoglio et al., 2011). An additional potential benefit is that it is suitable for vegetarians. Regarding the contraction-dependent pathway and building up on results by Weber-Carstens et al. we additionally will test whether GLUT4 translocation can be fostered by neuromuscular electrical stimulation in the Hephaistos immobilisation model.

A further aspect of the proposed study is related to rehabilitation, which is critical in astronauts after enduring space flights. Many systems of the human body including the central nervous system require extensive physical training for complete rehabilitation. It seems possible to facilitate rehabilitation with neurophysiological approaches that have already been demonstrated to promote neural activity. One of the techniques is anodal transcranial direct current stimulation (tDCS) that was shown to improve motor learning and consolidation (i.e. positively affect long-term motor memory). In the present study, tDCS is applied in the recovery phase after immobilization, while subjects are performing a physical training intervention. Therefore, the present study will test whether tDCS may improve learning after longer-term immobilization.

2.1.1 The Objectives of the Proposed Study

1) Primary Objective:

To test whether insulin sensitivity and glucose uptake in immobilized calf muscles can be improved by dietary supplementation of lupine seeds and neuromuscular stimulation in immobilized soleus and gastrocnemius muscle.

2) Secondary Objectives:

a) To assess in how far immobilization-induced reductions in muscle mass and bone mass can be mitigated by dietary supplementation of lupine seed and neuromuscular stimulation

b) To assess whole-body glycemic effects of the interventions.

c) To assess the application of transcranial direct current stimulation (tDCS) on rehabilitation (physical training) after immobilization.

2.1.2 The Hephaistos Orthosis

The Hephaistos orthosis was conceptualized at the German Aerospace Center in cooperation with the German Sport University and finally produced by Ortema, a company for orthopedic devices. The Aeromedical institute of the German Aerospace center is certified according EN ISO 13485:2003 + AC:2007 for designing and manufacturing of medical devices for Aeronautic-, Space- and Traffic Medicine. Construction of the orthosis was performed by Ortema-Orthopädie-Technik, Markgröningen, Germany, The manufacturing of the *customized* orthosis is in accordance with the directive 93/42/EWG (Appendix 1). An exemplary declaration of conformity is attached to this ethical application; however, each implemented orthosis shall receive a unique declaration of conformity. With this orthosis it is possible to achieve a significant unloading of both the soleus and the gastrocnemius muscle in the lower leg while subjects are able to pursue their daily life. By a fixation of the ankle and the prevention of rolling the forefoot it is possible to significantly reduce mechanical forces acting on the soleus and gastrocnemius muscle in the lower leg while gravitational forces are not affected. In a first study implemented in 2011 and 2012 at the German Aerospace Center (HEP-Study, Ethical Application 2010169, Ärztekammer Nordrhein) the effects on muscle, bone, cartilage and blood vessels in

healthy male subjects were investigated during 56 days of wearing the orthosis. It could be shown that gravitational forces alone are not sufficient enough to maintain muscle and bone mass.

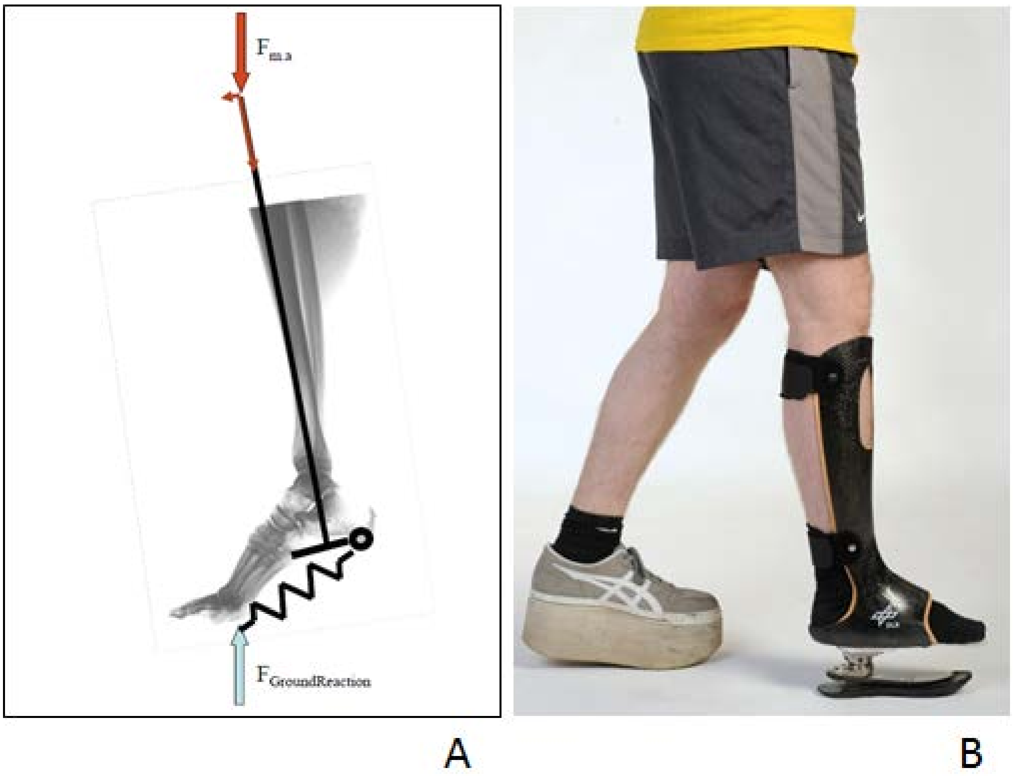

Figure 1: *The Hephaistos Orthosis.* (A) Free Body Diagram of the Hephaistos Orthosis. (B) Subject wearing the Hephaistos Orthosis.

Before the start of the intervention phase the subjects will be advised how to use the orthosis and they will learn to carry out their daily activities, including normal gait and stair negotiation with the orthosis during several training sessions at the DLR.

2.1.3 Lupine Seeds

The lupine flower belongs to the legume family (Fabaceae). Its seeds are eight to fourteen millimeters long and very rich in protein. These seeds have been commonly known since long, in particular around the Mediterranean. However, relatively little is known from a scientific background. Over the last years the group of Marcello Duranti (Milan, Italy) has focused on a special glycoprotein of the white lupin – the γ-

Conglutin. In early studies the group has tested the influence of γ-Conglutin on blood glucose levels in rats and glucose consumption in HepG2 cells (Lovati et al., 2011), to then assess the effect in healthy human subjects (Bertoglio et al., 2011). Regardless, the exact mechanism remains unclear and it remains unknown whether γ-Conglutin *per se,* or rather other constituents of the seeds are responsible for the glucose lowering effect. Therefore, whole lupine seeds or lupine flour will be applied in this study. Since Bertoglio et al. (2011) and Lovati et al. (2011) could show that γ-Conglutin seem to play an important role, the γ-Conglutin content of the seed or the flour will be tested before the study. The acute response of lupine protein on blood glucose and serum insulin concentrations will be tested in a pre-study at the DLR (NutriHEP pre-study). The study design and implementation of the pre-study is presented in 2.5.

2.1.4 Neuromuscular Electrical Stimulation (NMES)

The positive effects of neuromuscular electrical stimulation are widely appreciated. Thus, electrical stimulation was shown to have a positive effect on the translocation of the glucose transporter type 4 (GLUT4) in intensive care unit patients (Weber-Carstens et al., 2013). In this study we want to apply electrical muscle stimulation on the muscles of the lower leg to improve the glucose uptake in the muscle. The exact protocol for the neuromuscular electrical stimulation is listed under 2.7 – electrical muscle stimulation. The subjects will be trained on how to attach the electrodes correctly to the muscles and how to connect and use the device before the start of the intervention period in several sessions at the DLR.

2.1.5 Anodal transcranial direct current stimulation (tDCS)

As mentioned earlier, it is foreseen to apply tDCS in order to test its effect on rehabilitation (physical training) after immobilization. The protocol of this part of the study is described below (see 2.8) and will be offered as an optional support during rehabilitation.

2.2 Study Medication / Dosage Form

Not applicable.

2.3 Baseline Data Collection Pharmacological-Toxicological Testing

Not Applicable.

2.4 Type of the Study

Epidemiological study.

2.5 Nutritional Pre-Study to test the Acute Effect of Lupine Flour in Comparison to Whey Protein

Although whey proteins have favorable effects against the muscle atrophy response, they also have an acid-forming character in long-term consumption, which is negatively affecting protein metabolism. Such an effect has not been observed in lupine proteins so far, therefore the acid-base balance will be analyzed additionally, and we thus expect lupine proteins to be superior to whey proteins for skeletal muscle. However, there has not been a like-for-like comparison of the dietary effects of the two. Hence, we wish to organize a nutritional pre-study for NutriHEP in order to examine the acute effects of lupine seed proteins, since we plan to use them as dietary component in the NutriHEP study. Whey proteins, which are known for their insulinotropic effects and which can reduce the postprandial glycemia in healthy subjects as well as in subjects with type 2 diabetes (Frid et al., 2005), will be used as reference condition. The pre-study will test for non-inferiority of lupine proteins to whey proteins. To that purpose, the pre-study will focus on the physiological responses to a standardized meal with a high glycemic index on both the postprandial blood glucose level and the serum insulin. The outcomes of the study can thus affirm suitability of lupine seed in the current scenario, and it is expected that lupine proteins have an even better efficiency than whey proteins, without a negative secondary action and could act as a proper substitute in cases where whey

proteins cannot be used in the presence of dietary reasons or nutritional preferences (e.g. vegan or allergy).

Hypotheses

A) Lupine proteins lower the postprandial blood glucose level equivalent (± 5 %) to or to a greater extent than whey proteins.
B) Lupine proteins have an equivalent (± 5 %) or a stronger insulinotropic effect than whey proteins.
C) Lupine proteins have a lower effect on the acid-base balance than whey proteins.

Study design

The study will be conducted in a balanced crossover design. It will involve twelve healthy female and male subjects. The study encompasses one visit for the baseline data collection followed by three visits for the interventions (as shown in Figure 4) and a short visit on every subsequent day, within a total duration of two weeks. To standardize the dietary intake, subjects will receive a standardized breakfast, and then a standardized lunch test meal that is rich in carbohydrates and has been supplemented with either lupine or whey protein, or which contains no additional protein but is calorically balanced with both types of test meals. The dinner is standardized likewise. Immediately before and at several time points after ingestion of the test meals, blood samples will be drawn to determine the endpoint variables, namely blood glucose and serum insulin levels. In the morning, the afternoon and the subsequent morning arterial blood gas (ABG) will be measured. For the meals and the blood withdrawals the following scheme will be applied:

Exemplary Study Day	
**Time**	
07:45am	meeting at the Institute of Aerospace Medicine
08:00am	application of the venous catheter + 1st arterial blood gas
08:30am	1st baseline + standardized breakfast
	break (approx. 4 h, no food intake)
12:30am	2nd baseline + standardized test meal (+?)
12:42am	test meal finished (T_0)
12:52am	1st blood withdrawal (T_0 + 10)
01:02pm	2nd blood withdrawal (T_0 + 20
01:12pm	3rd blood withdrawal (T_0 + 30)
01:22pm	4th blood withdrawal (T_0 + 40)
01:42pm	5th blood withdrawal (T_0 + 60)
02:42pm	6th blood withdrawal (T_0 + 120)
03:42pm	7th blood withdrawal (T_0 + 180) + removal of the venous catheter
03:45pm	2nd arterial blood gas
04:00pm	end of examination
	dinner
**Subsequent day**	
09:00am	3rd arterial blood gas

Figure 2: *Exemplary study day for the subjects.*

The subjects will be asked to finish their test meals within twelve minutes. After consuming the meals, subjects will be asked to abstain from any other ingestion the whole examination day, except for about 1.5 L of water. During the experiment (until 4 pm) subjects will also be asked to avoid physical activity but can move in a normal

pace (sitting, walking, etc.). There will be a break of at least 24 hours between testing days to avoid any interference between the different interventions (as shown in Figure 4). In addition to that subjects will be randomly assigned to a partly balanced sequence plan of conditions (as shown in Figure 3). All testing will take place at the Institute of Aerospace Medicine at the German Aerospace Center (DLR) in Cologne.

Subject	1st Visit	2nd Visit	3rd Visit
1	A	B	C
2	C	B	A
3	B	A	C
4	C	A	B
5	A	C	B
6	B	C	A
7	A	B	C
8	C	B	A
9	B	A	C
10	C	A	B
11	A	C	B
12	B	C	A

A = lupine protein
B = whey protein
C = no additional protein

Figure 3: *Sequence plan for the protein supplementation per visit.* To create a balanced randomization and avoid interferences, the supplementation is evenly distributed among the subjects. For example: Subject 1 will eat lupine protein (A) at the first visit, whey protein (B) on the second visit and no additional protein (C) on the third visit (see also Figure 4). Subject 2 will complete the same sequence in reverse order.

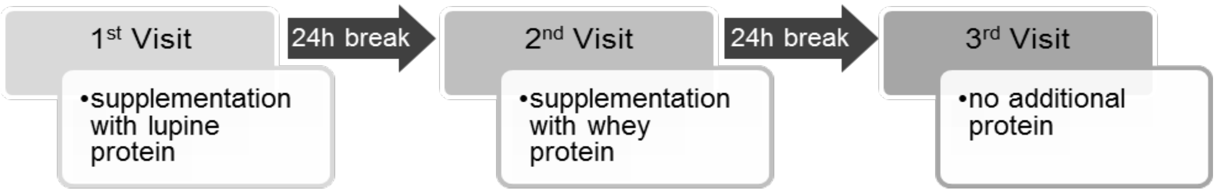

Figure 4: *Exemplary supplementation sequence for Subject 1.*

Protocol and Measurements

Inclusion and exclusion criteria and baseline data: applicants will be screened by interview and physically examined by a medical doctor. A 9.6 ml blood sample, will be obtained to assess fasting blood glucose, the glycated hemoglobin (HbA1c), a haemogram and for a clinical chemical examination (albumin, alkaline phosphatase, alanine aminotransferase, aspartate aminotransferase, bilirubin, creatinine, cholesterol, creatine kinase , GGT, HDL, LDH, LDL, lipase, triglycerides, calcium, chloride, magnesium, phosphate, potassium, sodium, protein, uric acid). Moreover

the subjects will be asked to obtain a urine sample for a urine test strip. Two additional tests, HIV and hepatitis B and C test, will be carried out in order to reduce the risk of infection for the medical doctors involved in the study. The subjects will be informed if there are some positive test results, which mean hepatitis infection or HIV infection.

Testing visits: Study subjects will arrive in the laboratory after overnight fasting. A venous catheter will be placed, a first baseline blood withdrawal will be taken and the 1st arterial blood gas conducted. Four hours after the breakfast a second baseline is drawn and the subject will ingest the test meal. Once this is finished (T_0) blood samples will be drawn at T_0 + 10, 20, 30, 40, 60, 120 and 180 minutes (as shown in Figure 2). In all this blood samples the serum insulin and blood glucose parameters will be measured. After T_0 + 180 min the 2nd arterial blood gas is taken. The third one will be examined in the morning of the subsequent day.
Blood taken for the arterial blood gas is capillary blood from the finger tip. A precise prick with a needle leaves a blood drop to exit the vessel and taken up into a glass capillary under conditions of the capillary effect. The blood gas analyzer then determines all defined parameters (pH, pCO_2, pO_2, HCO^{3-}, base excess) automatically. Because the volume of the glass capillary amounts only 85 µl this blood sample is not included in the total. Over the three study days a total of 148.5 ml blood will be drawn from each subject.

2.6 Study Implementation

After completion of the nutritional pre-study, the main part of the NutriHEP study can be implemented. It will involve 24 male ambulatory test subjects. All measurements and experiments will be performed either in the physiology laboratory of the German Aerospace Center, Cologne, or in the new medical research facility :envihab of the German Aerospace Center, Cologne.. The intervention time (INT) lasts 60 days. As shown in table 1, baseline data collection will begin four weeks prior to the start of the intervention time (Base line data collection, BDC). The study will be ambulatory so that the subjects are able to pursue their habitual daily activities. After completion of the INT-phase, there will be a 2-week follow-up to study

the recovery of alterations (recovery phase, R). Optionally, in addition to the normal follow-up, subjects will be offered a neurophysiological training called “anodal transcranial direct current stimulation” (tDCS) to improve rehabilitation.

The 24 study subjects will be divided into two groups, each consisting of 12 subjects. One group will represent the control group wearing the only orthosis, and the other group will receive dietary supplements and electrical muscle stimulation in addition to wearing the orthosis. The side of the intervention will be chosen by tossing a coin. Twenty-eight days before the intervention period several methods focusing on muscle and neurophysiological behavior will be performed (see table 1). Twenty-seven days before the intervention period (BDC-27) biopsies from the intervention legs’ soleus and gastrocnemius muscle will be taken. This time period was chosen to decrease the risk of thrombosis. In addition to muscle biopsies intra-muscular microdialysis, a hyperinsulinemic-euglycemic clamp and a pQCT measurement will be performed on twenty-six days before the intervention period (BDC-26), as well as the basal metabolic rate will be determined. Before, during and after the study blood samples will be taken at ten times. At these visits (except for the first one on BDC-28), the subjects will receive a defined diet for two days. Three out of the ten blood samples will be taken before (BDC-28, BDC-2, BDC-1) and two shortly after the intervention time (R+2, R+14). The remaining five blood samples will be taken at day 4, day 14, day 28, day 42 and day 60 of the intervention time (INT4, INT14, INT28, INT42, and INT60). On the day before the end of the intervention period biopsies will be taken again from the intervention legs’ soleus and gastrocnemius muscle (INT59). At the last day of the intervention period (INT60) an intra-muscular microdialysis and a hyperinsulinemic-euglycemic clamp will be performed. pQCT measurements will be performed at intervention days INT4, INT28 and INT59, as well as in the recovery phase on day R+14 at the DLR. An overview of the measurement schedule is given in the Table below.

As mentioned above anodal transcranial direct current stimulation (tDCS) will be applied during the rehabilitation phase to test whether this method can improve learning after a long-term immobilization, if the subject chose to participate in the training. tDCS will then be applied four times before the intervention period (BDC- 6, BDC-4, BDC-2 and INT1 before applying the orthosis) and nine times after the intervention period (R+2, R+4, R+6, R+8, R+10, R+12, R+14, R+16 and R+30).

Between the several measurements there has to be a break of at least 24h, between the eighth and the ninth measurement there has to be a break of two weeks.

	Recruitment	BDC-28	BDC-27	BCD-26	BDC-2	BDC-1	INT1	INT4	INT14	INT28	INT42	INT58	INT59	INT60	R+2	R+14
Medical Examination	x															
Psychological Examination	x															
Training on how to use orthosis			45		60	20										
Training on how to use NMES device			30		30	30										
NMES Training								30		30						
Start Intervention							90									
Muscle Biopsies			45										45			
Microdialysis				360										360		
Clamp				360										360		
Basal Metabolic Rate			60										60			
pQCT			25					25		25			25			25
Accelerometer			60				60		60		60					
MRI		60										60				
MVC		15										15				
Jump Test		15										15				
Blood		20			20	20		20	20	20	20			20	20	20
EMG		30										30				
PNS		30										30				
TMS		30										30				
tDCS (optional)																
Mechanical Properties of Tendon and Aponeurosis		60										60				
Total [min]		**320**	**265**	**360**	**110**	**70**	**150**	**75**	**80**	**75**	**80**	**300**	**130**	**360**	**20**	**75**

Table 1: *Time Schedule for the NutriHEP-Study.* BDC: Baseline Data Collection, INT: Intervention, R: Recovery

2.7 The Introduction and Explanation to the Subjects

The purpose and the procedure of the proposed study will be orally explained to each subject before the study. In addition, each subject will receive following documents:

- The explanation and requirement of this study in written form (subject information)
- Subjects' consent;
- Subjects' contract;
- Privacy statement;
- Consent to examination of blood for HIV and hepatitis;
- Insurance documents;

The data collected in this study are stored in anonymous form. The names of the volunteers included in this study is replaced by random English letters (subject A, B, C, D, E and so on). It will thus be impossible for anybody not involved in this study to identify the subjects from whom the recorded data originate. At least two weeks before the measurements, a medical examination is performed for each subject at the Institute for Aerospace Medicine of DLR in order to ascertain good health, and the written informed consent will also be obtained on this occasion.

The medical examination will include clinical-chemical test (GT, lipase, Alkaline phosphatase, HbA1c), hematology test, blood clotting test, urinalysis test, resting ECG test, blood glucose test, thrombosis screening and medical history examination. Two additional tests, HIV and hepatitis B and C test, will be carried out in order to reduce the risk of infection for the medical doctors involved in the study. The subjects will be informed if there are some positive test results, which mean hepatitis infection or HIV infection.

2.8 The Methods Adopted in the Proposed Study

Neuromuscular Electrical Stimulation (NMES)

During the entire period of unloading/muscle silencing, subjects are asked to complete two daily sessions of NMES (one in the morning and one in the afternoon, separated by at least 8 hours), on all days of the week. NMES is delivered with a portable battery-powered constant-current stimulator (Model, Brand, Company - to be defined) connected to three self-adhesive electrodes. Two 5 x 5 cm electrodes are positioned over the motor point of gastrocnemius lateralis and gastrocnemius medialis muscles (Botter et al. 2011), and one 5 x 10 cm electrode is placed longitudinally on the proximal aspect of the soleus muscle. Biphasic rectangular pulses lasting 200 µs are delivered at a frequency of 30 Hz and with an on:off ratio of 5:10 s (both ramp-up and ramp-down of 1 s) for 20 min. Current amplitude (range 0-100 mA) is monitored on-line and is gradually increased during each session to the individual level of maximally tolerated intensity (depending on pain threshold). Each session is preceded by a standardized warm-up consisting of 5 min of submaximal (15-30 mA) and low-frequency (5 Hz) NMES. Subject are asked to complete the morning session in a seated position (with a knee angle of 90°) and the afternoon session in a standing position; in both cases, a resistance to plantar flexion evoked contractions is offered.

Muscle Biopsies

Tissue samples shall be obtained from the soleus and gastrocnemius by muscle biopsy. Tissue sampling will be done under sterile conditions and with local anaesthesia of the skin and fascia above the muscle using 2-3 ml of lidocain (1%). Of note, muscles do not contain nociceptors, and therefore infiltration of the skin and fascia suffices to prevent pain perception. As soon as the study subjects reports numbness of the infiltrated skin, a 12mm incision will be made into the skin and fascia, and a sample of approximately 150-200 mg will be obtained with a biopsy rongeur (Ferr. Smith, Fa. Zepf, Dürbheim). Alternatively, an Acecut spring-loaded biopsy needle can be used when the rongeur approach is not feasible. This, however, yields only 60mg of tissue, which will not be sufficient to carry out all planned tissue analyses.

Samples shall be obtained from the lateral gastrocnemius muscle, using a lateral approach at the mid belly, and likewise with a lateral approach from the soleus muscle, approximately 1 cm below the gastrocnemius. This way, the important nerve-vessel bundles receive protection by the fibula.

Once the tissue has been obtained, wounds will be closed by intracutaneous suture and covered with a sterile plaster. A compression bandage will be applied in order to reduce any after-bleeding. This should stay in place for up to 4 hours, and the sterile plasters shall remain for a minimum of 48 hours. During this period, subjects should also refrain from maximal efforts of the calf musculature. The foot pulse will be checked before test subjects leave, in order to ascertain patent perfusion of the lower legs.

There are two sets of biopsies planned, one during baseline collection and one after 57 days of immobilization. Taking a set of biopsies will last approximately 30 minutes. The muscle biopsies obtained will be used for sections as well as for protein and mRNA extractions. The sections will be stained immunehistochemically for GLUT4 to detect the GLUT4 position while protein and mRNA levels are analyzed to assess the two GLUT4 recruiting pathways.

Microdialysis

Organ and tissue perfusion and metabolism can be regulated at various levels, including enzymes, hormones and the autonomic nervous system. An established method for simultaneous *in situ* monitoring of changes in tissue perfusion and metabolism during physiological or pharmacological interventions on theses regulatory levels in readily accessible organs such as skin, adipose tissue and muscle is the microdialysis technique. Additionally, changes in interstitial levels of regulatory non-protein and protein-derived hormones can be monitored. The core part of this technique is a very thin (outer diameter 1 mm) double-lumen flexible probe, about 6 cm long, with an outer membrane semipermeable for molecules up to 20 or 100 kDa (depending on the probe used). This probe is inserted, for example, into the skeletal muscle (*Vastus lateralis* muscle) with a splittable introducer. The skin puncture site is determined as follows: measure the distance between the *Spina iliaca ant. sup.* and upper edge of the *Patella*, divide the distance into thirds, set a first mark at the border between the middle and distal third, ask the test subject to tense the thigh muscles, set a second mark about 4-6 cm lateral to the first mark -

depending on muscle volume - right in the middle of the *Vastus lateralis* muscle ball. Before inserting the probe, the skin area around the puncture site and the vicinity of the muscle soft connective tissue is anaesthetized by injecting subcutaneously about 2 ml of a lidocaine solution (Xylocitin® 1%, Jenapharm GmbH, Jena, Germany). For inserting a probe into the *Soleus* or *Gastrocnemius* muscle the procedure is adapted accordingly. After inserting the probe, the tissue is perfused at a rate of 2 µl/min with Ringer’s solution (E156®, Serumwerke Bernburg AG, Bernburg, Germany) enriched with 50 mM ethanol (Alkohol-Konzentrat 95%, B. Braun Melsungen AG, Melsungen, Germany). A “71 MD probe” and a “107 MD pump” (both from µDialysis, Stockholm, Sweden) will be used. During the perfusion, the perfusate (inflow) turns into the dialysate (outflow) which can be analysed for different marker metabolites (glucose, lactate, pyruvate, glycerol), biological effectors, and indicators for the local acid-base balance. Changes in muscle perfusion and metabolism can be deduced from changes in the [ethanol]outflow / [ethanol]inflow - ratio and in dialysate marker metabolite concentrations. Microdialysis will be performed after a 12 hour (overnight) fast in the beginning and at the end of the study campaign. Total duration of each microdialysis study is approximately four hours. Further information on microdialysis can be downloaded on www.mdialysis.com. Microdialysis will be performed twice during the study, once before the intervention period and once at the end of the intervention period.

Hyperinsulinemic Euglycemic Clamp

Subjects will remain fasting from 7:00pm in the evening before the clamp until the end of the clamp, which is approximately 4:00pm the day of the clamp.
During the clamp, one hand is placed in a so-called hot-box which will be warmed to an air temperature of 55°C, which allows sampling of arterialized venous blood. Insulin will be infused intravenously by means of a precision pump during the blood glucose adjustment phase and during the insulin infusion steps.

The clamp is consisting of three phases, namely the baseline period, the insulin infusion step 1 and the insulin infusion step 2. The baseline period will last 30 minutes in which blood glucose concentration is continuously measured and maintained by a variable glucose infusion. For sampling of arterialized venous blood and to measure the blood glucose concentration a hand or forearm is cannulated and placed in a thermo regulated box with a temperature of approximately 55°C

throughout the clamp procedure to reach an arterialization of the venous blood. A low dose of heparin solution (10,000 U/100ml saline) will be infused via a double lumen catheter. A second cannula is inserted in the forearm vein of the same arm to sample serial measurements of insulin and blood glucose and is kept open by a continuous 0.9% saline infusion. The clamp level will be set to a blood glucose concentration of 100 mg/dl. The rate of glucose delivery is adjusted in response to the measured blood glucose concentration. Blood samples for determining blood glucose and insulin concentrations will be drawn at regular intervals during the experiment.

The second phase, the insulin infusion step 1 (0.25 mU/kg/min) lasts 240 minutes during which insulin is infused at a rate of 0.25 mU/kg/min. The last 60 minutes of this phase are regarded as steady state period 1.

The third phase, the insulin infusion step 2 (1.0 mU/kg/min) lasts another 240 minutes during which insulin is infused at a rate of 1.0 mU/kg/min. Again, the last 60 minutes of this phase are regarded as steady state period 2.

After this phase the clamp is stopped and subjects will be served a meal to neutralize any potential carry-over effect of the insulin infusion.

The total blood loss during on hyperinsulemic-euglycemic clamp will not exceed 37 ml.

Basal Metabolic Rate

The basal metabolic rate will be determined on the four weeks before the start of the study by indirect calorimetry. The measurement procedure (about 30 minutes long) takes place before breakfast. The principle of the method is based on a continuous measurement of oxygen uptake and carbon dioxide production. To this purpose, a spiroergometer will be used (CORTEX, Germany). Fresh and expired air are continuously analyzed for their O_2 and CO_2 levels, and the O_2 consumption and CO_2 production is determined. Not only changes in energy turnover but also the rate of oxidation of carbohydrates and fats can be calculated from these parameters with suitable mathematical models. The test subject is supplied with sufficient oxygen at all times.

Bone densitometry

Bone mineral content of the tibia shall be assessed by peripheral quantitative computed tomography (pQCT). This is an x-ray based imaging method that allows measurement of true volumetric density in cross sectional images, and it is therefore also able to assess bone geometrical information and derived predictors of bone strength. Two different devices shall be employed. Both of these devices rely upon a so-called scoutview that yields an identifiable reference line from a 2-dimensional overview of the tibia epiphysis. **Firstly**, conventional pQCT shall be performed with an XCT 3000 (Stratec Medical, Pforzheim, Germany). This device has been used in the majority of bed rest studies in the past, and it has also been used in the original HEP study. The data obtained with the XCT will therefore allow like-for-like comparison with the host of existing data. In order to be compatible with the scanning protocols in past studies, it is planned to obtain scans of the 4% distal tibia site twice during baseline data collection, and on days INT28, INT60, R+14 and R+28 (total of 6 scans per subject, plus 6 scoutviews). Once during baseline data collection, and on day R+14, a number of 18 additional scans shall be obtained in steps of 5% of the tibia's length (total of 36 scans, plus 2 scoutviews). Secondly, high resolution pQCT measurements shall be obtained with Xtreme CT (Scanco, Brütisellen, Switzerland). This device has a resolution of 100 µm and is thus ideal to discern bone losses from the cortical and trabecular compartments. Stacks of images shall be obtained from both the distal and the proximal tibia once during baseline data collection and on day R+14, *i.e.* when bone losses are expected to peak. All measurements shall be carried out in the physiology lab of the DLR institute for aerospace medicine.

The expected radiation dose for each XCT scan of the tibia is 0.00039 mSv for cross-sectional images, and 0.00017 mSv for the scoutview. Thus, all foreseen XCT-measurements comprise a total of (6+36)*0.00039 + (6+2)*0.00017 mSv = 0.01774 mSV. For the Xctreme CT measurements, the radiation dose is 0.0068 mSv per stack, which yields a total measurement dose of 4*0.0068 mSv = 0.0272 mSv. We expect that 10% of all images have to be repeated for reasons such as movement artefacts. Accordingly, the total radiation dose as per this study is expected to amount to 1.1 * (0.01774+0.0272) mSv = 0.049434 mSv. This dose is well below the 0.1 mSv which is commonly regarded as the threshold for negligible exposure to radiation.

Approval for the application of radiation for this study will be sought from the Federal Office for Radiation Protection (Bundesamt für Strahlenschutz).

Magnetic Resonance Imaging (MRI)

Using ^{1}H-MRI we record 3D images with high spatial resolution to measure the volume loss of plantar flexor muscles.

^{23}Na-MRI is capable to measure changes of the extracellular space that is predominantly containing sodium as cation.

^{1}H-MRS measures in specifically localized voxels the tissue composition of muscles. In this study we focus on atrophy related accumulation of fatty acids in the muscle fibers and a resulting relative loss in cell water of muscle fibers labeled by total creatin content.

The magnetic resonance technology summarizes non-invasive techniques for depicting and surveying human organs and assessing their chemical composition. Images or spectra are obtained by the interaction of hydrogen in body fat and water in a strong, constant magnetic field (3 Tesla) with radio frequency radiation (^{1}H: 128 MHz, ^{23}Na: 33 MHz) and rapidly switched magnetic field gradients. In this study we focus on the lower leg and the anatomical and physiological effects of the Hephaistos orthosis on plantar flexor muscles. Changes in muscle size are assessed by ^{1}H-MRI, the relative extracellular fluid content is determined by the sodium ion using ^{23}Na-MRI, and finally ^{1}H-MRS measures in specifically localized voxels the tissue composition of muscles. In this study we focus on fatty acid and total creatin content. The examinations will be carried out at rest and in supine position. The test subject will be asked to rest for 20 min before the first scan in order to wait for a constant distribution of the blood volume after lying down. This time is used for the acquisition of scout images and the planning of measurements. To avoid motion and to achieve a defined length of the calf musculature, the right foot is held in a rigid pedal at a comfortable angle of 70º to the couch. The total testing time will amount to approximately 60 min in the magnet of the machine for the preparation and the three examinations. The head is outside the tunnel of the magnet for the entire time. Ear guards will be offered to subjects in order to reduce the noise produced by the MRI scanner.

Maximal voluntary isometric contraction (MVIC)

MCV is measured before and after the intervention to determine the effects of wearing the orthosis on voluntary force development of the plantar flexor muscles.

The determination of MVC uses a standardised procedure for measuring the current maximum voluntary muscle strength at given angles of joints, i.e. muscle lengths. In this study, we determine the MVC of plantar flexor muscles using a pedal with integrated torque sensor. Subjects are tested in supine positions with extended knees and the foot held in neutral position (foot sole 90° to the tibia). MVC is determined as the highest torque reached in three trials of 5 seconds each, with 60 s recovery between trials. MVC is determined before and after the INT phase. However, subjects will be asked to practice this test two or three times before valid data can be acquired.

Vertical Jump Test

The aim of the test is to determine the neuromuscular performance and the power output of the lower extremities. To perform the test, subjects stand on a Leonardo force platform (Novotec Medical, Pforzheim, Germany) with hands placed on the hips. When the ground reaction forces indicate stable starting conditions, the integrated Leonardo software will automatically issue a signal, and subjects will be asked to perform a brief countermovement (*i.e*. squat), and then jump into the air as high as possible. The test is repeated until three successful jumps have been recorded. The jump height, as well as peak force and peak mechanical power are automatically analysed by the integrated Leonardo software. In order to ensure the participant's safe landing after the jump, a supervisor is positioned directly behind the jumping plate. This person is only allowed to touch the participant if a safe landing cannot be guaranteed. Every jump in which the supervisor is involved or the test subject lands beside the plate must be repeated.

Blood Sampling

Blood sampling

Fasting morning blood will be taken on ten study days (BDC-28,BDC-2, BDC-1, INT4, INT14, INT28, INT42, INT60, R+2, R+14) under standardized condition with the Sarstedt blood drawing system by a medical doctor or a phlebotombist. The

following parameters will be analyzed in order to get detailed insight into glucose-, calcium- and bone metabolism.

Glucose metabolism

Glucose

Blood glucose, commonly also called blood ‘sugar’ level is the amount of glucose (sugar) present in the blood of a human or animal. The body naturally tightly regulates blood glucose levels as a part of metabolic homeostasis.

Insulin

Insulin is a peptide hormone, produced by beta cells of the pancreas, and is central to regulating carbohydrate and fat metabolism in the body. Insulin causes cells in the liver, skeletal muscles, and fat tissue to take up glucose from the blood. With the exception of the metabolic disorder diabetes mellitus and metabolic syndrome, insulin is provided within the body in a constant proportion to remove excess glucose from the blood, which otherwise would be toxic. When control of insulin levels fails, diabetes mellitus can result.
Glucose and Insulin concentration will be used to calculate the so called HOMA Index. The homeostatic model assessment (HOMA) (=Insulin (µU/ml) x Glucose (mmol/l) / 22.5) is a method used to quantify insulin resistance and beta-cell function. It was first described under the name HOMA by Matthews et al. in 1985.

HbA1c

Glycated hemoglobin or glycosylated hemoglobin (HbA1c) is a form of hemoglobin that is measured primarily to identify the average plasma glucose concentration over prolonged periods of time. It is formed in a non-enzymatic glycation pathway by hemoglobin's exposure to plasma glucose. Normal levels of glucose produce a normal amount of glycated hemoglobin. As the average amount of plasma glucose increases, the fraction of glycated hemoglobin increases in a predictable way. This serves as a marker for average blood glucose levels over the previous months prior to the measurement.

Proinsulin

Proinsulin is produced in the pancreatic beta-cells and is normally further processed to insulin and C-peptide. It is only seen in low concentrations in the plasma of healthy subjects. An increase in the insulin demand, as provided by insulin resistance in later stages of type 2 diabetes mellitus, can result in increased expression of proinsulin into the blood. In clinical practice, fasting morning intact proinsulin can be used as highly specific indicator of clinically relevant insulin resistance.

Fructosamine

Fructosamine is a compound that can be considered the result of a reaction between fructose and ammonia or an amine. Fructosamines formed from serum proteins such as albumin are known as glycated serum protein (GSP), and are used to identify the plasma glucose concentration over time and so assess diabetic control over an intermediate period of time

1,5 Anhydroglucitol

1,5-anhydroglucitol (1,5-AG) is a validated marker of short-term glycemic control. It is a metabolically inert polyol that competes with glucose for reabsorption in the kidneys. Otherwise stable levels of 1,5-AG are rapidly depleted as blood glucose levels exceed the renal threshold for glucosuria. 1,5-AG more accurately predicts rapid changes in glycemia than hemoglobin A1C (A1C) or fructosamine. It is also more tightly associated with glucose fluctuations and postprandial glucose. Thus, 1,5-AG may offer complementary information to A1C.

GLP-1

Glucagon-like peptide-1 (GLP-1) is derived from the transcription product of the proglucagon gene. The major source of GLP-1 in the body is the intestinal L cell that secretes GLP-1 as a gut hormone. GLP-1 secretion by ileal L cells is dependent on the presence of nutrients in the lumen of the small intestine. The secretagogues (agents that cause or stimulate secretion) of this hormone include major nutrients like carbohydrate, protein and lipid. Once in the circulation, GLP-1 has a half-life of less than 2 minutes, due to rapid degradation by the enzyme dipeptidyl peptidase-4. It is a potent antihyperglycemic hormone, inducing glucose-dependent stimulation of insulin secretion while suppressing glucagon secretion. Such glucose-dependent action is

particularly attractive because, when the plasma glucose concentration is in the normal fasting range, GLP-1 no longer stimulates insulin to cause hypoglycemia. GLP-1 appears to restore the glucose sensitivity of pancreatic β-cells, with the mechanism possibly involving the increased expression of GLUT2 and glucokinase. GLP-1 is also known to inhibit pancreatic β-cell apoptosis and stimulate the proliferation and differentiation of insulin-secreting β-cells. In addition, GLP-1 inhibits gastric secretion and motility. This delays and protracts carbohydrate absorption and contributes to a satiating effect.

Adiponectin/Leptin

Adiponectin is a protein hormone that modulates a number of metabolic processes, including glucose regulation and fatty acid oxidation. Adiponectin is exclusively secreted from adipose tissue into the bloodstream and is very abundant in plasma relative to many hormones. Levels of the hormone are inversely correlated with body fat percentage in adults. The hormone plays a role in the suppression of the metabolic derangements that may result in type 2 diabetes, obesity, atherosclerosis, non-alcoholic fatty liver disease and an independent risk factor for metabolic syndrome. Adiponectin in combination with leptin (a protein hormone that plays a key role in regulating energy intake and energy expenditure, including appetite/hunger and metabolism. It is one of the most important adipose-derived hormones) has been shown to completely reverse insulin resistance in mice.

The blood amount needed for analyzing the parameters of glucose metabolism will be 7 ml per blood draw and therefore 63 ml (7 ml * 9 blood draws) for the whole study.

Calcium- and bone metabolism

Total calcium, ionized calcium

Calcium homeostasis is the mechanism by which the body maintains adequate calcium levels. Derangements of this mechanism lead to hypercalcemia or hypocalcemia, both of which can have important consequences for health. The serum level of calcium is closely regulated with a normal total calcium of 2.2-2.6 mmol/L (9-10.5 mg/dL) and a normal ionized calcium of 1.1-1.4 mmol/L (4.5-5.6

mg/dL). The amount of total calcium varies with the level of serum albumin, a protein to which calcium is bound. The biologic effect of calcium is determined by the amount of ionized calcium, rather than the total calcium. Ionized calcium does not vary with the albumin level, and therefore it is useful to measure the ionized calcium level when the serum albumin is not within normal ranges, or when a calcium disorder is suspected despite a normal total calcium level.

PTH

PTH (Parathyroid hormone) is biosynthesized in the parathyroid gland as a preproparathyroid hormone, a larger molecular precursor consisting of 115 amino acids. In healthy individuals, regulation of parathyroid hormone secretion normally occurs via a negative feedback action of serum calcium on the parathyroid glands. Intact PTH is biologically active and clears very rapidly from the circulation with a half-life of less than four minutes. PTH undergoes proteolysis in the parathyroid glands, but mostly peripherally, particularly in the liver but also in the kidneys and bone, to give N-terminal fragments and longer lived C-terminal and Mid-region fragments. Intact PTH assays are important for the differentiation of primary hyperparathyroidism from other (nonparathyroid-mediated) forms of hypercalcemia, such as cancer, sarcoidosis and thyrotoxicosis. The measurement of parathyroid hormone is the most specific way of making the diagnosis of primary hyperparathyroidism. In the presence of hypercalcemia, an elevated level of parathyroid hormone virtually establishes the diagnosis.

25, OH Vitamin D, 1,25 OH Vitamin D

Vitamin D is a steroid hormone involved in the intestinal absorption of calcium and the regulation of calcium homeostasis. Physiological Vitamin D3 levels result not only from dietary uptake but can also be produced from a cholesterol precursor, 7-dehydrocholesterol, in the skin during sun exposure. In the liver, the vitamin is hydroxylated to 25-hydroxyvitamin D (25-OH-Vitamin D), the major circulating metabolite of Vitamin D. Although 1,25-(OH)2 Vitamin D portrays the biological active form of Vitamin D, which is synthesized in the kidney, it is widely accepted that the measurement of circulating 25-OH-Vitamin D provides better information with respect to patients Vitamin D status and allows its use in diagnose hypovitaminosis. The concentration of 25-OH-Vitamin D decreases with age and a deficiency is common

among elderly persons. Clinical applications of 25-OH-Vitamin D measurements are the diagnosis and therapy control of postmenopausal osteoporosis, rickets, osteomalacia, renal osteodystrophy, pregnancy, neonatal hypocalcemia and hyperparathyroidism

Bone formation marker bone specific alkaline phosphatase (bAP) and Procollagen type 1 N-terminal propeptid (P1NP)

The skeletal, or bone-specific, isoform of alkaline phosphatase is a tetrameric glycoprotein found on the surface of osteoblast cells. Osteoblasts are a type of cell responsible for laying down the protein matrix of bone, which calcium salts (particularly phosphates) are deposited. It's been shown to be a biochemical indicator of bone turnover.

P1NP is the preferred marker for bone formation. It is a specific indicator of type 1 collagen deposition. It is released as a trimeric structure, but degrades to a monomer. It is increased in states of high bone turnover and hence may be of value for assessing the response to treatment of osteoporosis and Paget's disease.

Osteocalcin

Osteocalcin is secreted solely by osteoblasts and thought to play a role in the body's metabolic regulation and is pro-osteoblastic, or bone-building, by nature. It is also implicated in bone mineralization and calcium ion homeostasis. Osteocalcin acts as a hormone in the body, causing beta cells in the pancreas to release more insulin, and at the same time directing fat cells to release the hormone adiponectin, which increases sensitivity to insulin

Sclerostin

Sclerostin is the protein product of the SOST gene. The highest expression of sclerostin throughout the adult skeleton has been observed in hypertrophic chondrocytes and osteocytes.Sclerostin blocks canonical Wnt signaling by binding to the Wnt coreceptors LRP5/6, inhibiting bone formation by regulating osteoblast function and promoting osteoblast apoptosis. Sclerostin also antagonizes bone morphogenetic protein (BMP) action (e.g. osteoblast differentiation), but does not inhibit direct BMP-induced responses.Sclerostin expression is down-regulated by Parathyroid hormone (PTH), as well as, by the mechanical stimulation of bone.

Reduced expression of sclerostin can result in van Buchem disease, while a complete absence results in Sclerosteosis. Patients affected by Sclerosteosis show progressive hyperostosis and sclerosis of the skull, mandible and all long bones. Bone mineral density (BMD), bone volume, bone formation rate, and bone strength are significantly increased, while overall skeletal morphology appears to be normal.

DKK1

Dickkopf-1(DKK-1) is a 28,672 Da secreted protein that acts as soluble inhibitor of the WNT signaling pathway. This pathway contains lipid–modified glycoproteins that activate cell surface receptor-mediated signal transduction to regulate cell activities like: cell fate, proliferation, migration, polarity and gene expression. DKK-1 regulates different developmental processes and is also involved in the regulation of bone metabolism as it inhibits the differentiation of osteoblast.

The blood amount needed for analyzing the parameters of glucose metabolism will be 9 ml per blood draw and therefore 81 ml (9 ml * 9 blood draws) for the whole study.

Whole blood draw amount:

Medical screening: 20 ml

Parameters of glucose metabolism: 63 ml

Clamp test: 74 ml

Parameters of calcium and bone metabolism: 81 ml

Whole blood amount: 238 ml

Standardized Diet

Since glucose-, calcium- and bone metabolism are highly influenced by nutrition, diet will be standardized 2 days prior to the blood sampling days. Individual menu plans will be established according to subjects´ body weight and will be kept constant for energy, protein, fat, carbohydrates, calcium, sodium and water. Minerals and vitamins will reach the daily recommended intake levels according to “Dietary Reference Intake” (DRI, National Academy of Sciences. Institute of Medicine. Food

and Nutrition Board, USA). The food will be prepacked and handed over to the test subjects.

Standardized diet will be given on the following study days:
BDC-4,-3,-2,-1, INT2,3 ,12,13, 26,27, 40,41, 58,59,, R+0,+1, R+12,+13

Accelerometer

The Hephaistos device will be equipped with accelerometers that record 3-dimensional linear acceleration in order to document usage of the Hephaistos. In addition, subjects will also be asked to wear accelerometers on their waist in order record acceleration near the center of mass, a surrogate measure of ground reaction forces. For both purposes, commercially available will be used (Gulf Data Concepts, Tampa, USA). Due to storage limitations, both accelerometers have to be replaced on a weekly base.

Electrophysiological techniques applied before and after immobilization and during rehabilitation

Electromyography

Surface electromyographic electrodes (Blue sensor P, Ambu, Bad Nauheim, Germany) will be attached with surgical tape to the skin over the soleus, gastrocnemius and tibialis anterior muscle. Electrodes will be placed over the muscle belly. All recording cables connected to the subjects will have an isolated ground supply in order to guarantee that no electrical shock will be applied.

Peripheral nerve stimulation (PNS)

N. tibialis will be excited using a commercially-available stimulator (Digitimer DS7a, Digitimer Ltd., UK). The stimulation occurs with surface electrodes (Blue sensor P, Ambu, Bad Nauheim, Germany) attached to the skin with surgical tape. The cathode will be placed directly on the skin above the nerve. Before the measurements start the cathode will be moved over the skin until the best position for evoking a motor response resulting from electrical stimulation is found. The second electrode (anode) will also be fixed with surgery tape. The position of the anode is

not that important for achieving proper results, nevertheless care will be taken that its position will not affect electromyographic recordings.

The stimulator is a medically-rated component device that has been approved according to European standards (EN 61010-1 1995, EN 50082-1 1995). The stimulator has 3 built-in safety features to prevent harm to the subjects. First, there is a limit of the amount of energy that builds up in the stimulator so that overheating of the stimulator is prevented. Second, the maximum amount of current that is given as output from the stimulator is also limited. This limit can be selected by the experimenter to be between 1 and 100 mA. Third, the stimulator output is AC-coupled (alternating current) to prevent electrical burn of the tissue beneath the electrodes, which is a risk when direct current stimulation is applied.

The intensity of the stimulus will be controlled with respect to a multiple of the lowest perception threshold that is reported by the subjects.

Transcranial magnetic stimulation

Magnetic stimuli will be delivered to the primary motor cortex (M1) and the descending corticospinal tract at the cervicomedullary junction (cervicomedullar stimulation, CMS) by a commercially available magnetic stimulator (Magstim, Magstim Ltd., UK). The stimulation is delivered via a magnetic coil transducer that delivers a magnetic pulse, which in turn induces proportional current in the tissue to stimulate the nerve cells. The advantage of magnetic stimulation over classical direct percutaneous electrical brain stimulation (TES) is that electrical energy can cross the skin and bones without pain. Despite the different techniques, both magnetic stimulation and TES induce electrical currents to excite neural tissue. When using TMS, care must be taken that enough electrical current is delivered to excite neurons in the CNS. This can be achieved if the current that passes through the coil changes within some few hundred microseconds. If the magnetic field is strong enough and the threshold of the nerve cells is exceeded, an evoked potential can be found in the electromyographic recordings. TMS in the present study targets the plantarflexor muscles (m. soleus, m. gastrocnemius) of the lower (immobilized) leg.

Anodal transcranial direct currect stimulation (tDCS)

Anodal tDCS of M1 is used to promote rehabilitation in the present study (see below). The stimulation electrode will be placed on the scalp over M1 and the reference electrode over the contralateral supraorbital ridge. Stimulation intensity is set to 2 mA.

tDCS was developed in animal experiments. Here, inducing direct electrical currents with electrodes placed within the brain or on the skull caused a change of spontaneous neural activity (Bindman et al., 1964). Whereas anodal stimulation of the tissue resulted in an increase in spontaneous neural activity, cathodal stimulation resulted in a reduction in such spontaneous activity (Purpura & McMurtry, 1965). The mechanism for this change has been argued to be a modulation in membrane potential of the stimulated neurons (Nitsche et al., 2003).

The effect of direct current stimulation in humans was later first tested on changes in motor performance in a choice reaction task (Elbert et al., 1981), which was executed faster with anodal tDCS of M1. Later, anodal tDCS of M1 was shown to increase corticospinal excitability measured with single pulse TMS (Nitsche & Paulus, 2000). To evoke this effect, the strength of the electrical stimulation is set between 1 mA and 2 mA and the duration of the stimulation to approximately 10 – 20 min (for review see (Stagg & Nitsche, 2011)).

Experimental procedure

Each subject will participate in 4 experimental sessions, i) before immobilization, ii) after 8 weeks of immobilization, iii) after 2 weeks of rehabilitation (i.e. post immobilization), and iv) after 4 weeks of immobilization. Measurements will be carried out at rest, while subjects execute brief ballistic and tonic (isometric) contractions of the plantarflexor muscles of the immobilized leg and additionally during a training protocol during rehabilitation.

Additionally, there is an optional possibility for the subjects to participate in rehabilitation training. The rehabilitation training includes 13 sessions á 20min, 4 before (baseline), 8 after immobilization (recovery), and 1 after 2 weeks after the recovery period (consolidation). Training is performed every second day (i.e. training starts approximately 8 days before immobilization, and over a time period of 18 days directly after immobilization). The training protocol during rehabilitation consists of a sequence learning tasks that is applied similar to (Reis et al., 2009). Instead of using the finger in the study of (Reis *et al.*, 2009) the foot is used in the present study.

During rehabilitation training, tDCS will be applied in half of the tested subjects.

The following neurophysiological techniques will be applied (all methods are described above): Single pulse TMS, conditioned H-reflexes using TMS of M1 and CMS, paired pulse TMS (using combined sub- and suprathreshold stimulation, ISI 2.5 ms and ISI 10 ms) (Rothwell *et al.*, 2009).

Portable ergometer

The portable ergometer (Figure 2) is composed by an adjustable chair taking into account subject anthropometric characteristics. A foot plate is linked to the chair and allows adjusting knee as well as ankle angles based on position requirements. A shoe is firmly fixed on the foot plate and is adapted to the foot size of the subject. The axis of rotation of the ankle will be adjusted to the center of rotation of the foot plate. A tension-compression load cell (TEDEA 614 "S" Type) is attached to the extremity of the foot plate to get torque data. The foot plate is linked to the axis of rotation to an optical singleturn encoder 13 bit (GA241 (IVO)) allowing investigating angular displacement. An 8.5w holding magnet (Mécalectro 58201) is linked to the tension-compression load cell and allows quick-release movement when deactivated. During testing, the subjects will sit on the ergometer previously adapted to the subject anthropometric characteristics so that 1) the knee joint angle will be 120° (180° being full extension), 2) the ankle joint angle will be 90° (i.e., neutral position) and 3) the lateral malleolus will coincide with the axis of rotation of the footplate. Dorsal support will be provided during the rest periods, but not during voluntary contractions, to prevent extraneous movements and to limit the contribution of other muscles such as the trunk extensor muscles. The investigated foot will be firmly strapped to the footplate of the actuator. Except for passive range of motion test, all tests with this ergometer will be performed with previously defined joint angles.

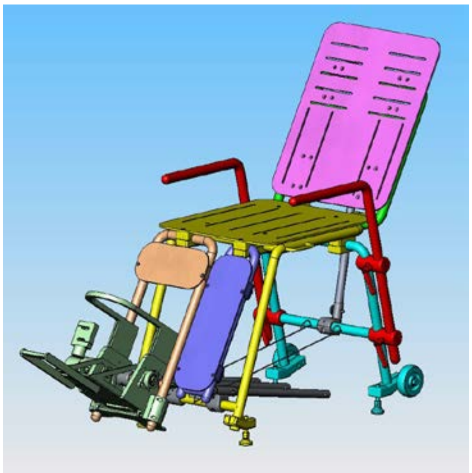

Figure 2: Portable ergometer

DESCRIPTION OF THE EXPERIMENTS

Passive Range of Motion

The passive range of motion (ROM) is the largest possible movement of the foot in extension and flexion when the foot is manipulated by an operator; the subject is totally passive. The subject shall stay relaxed and not doing anything except telling the operator to stop when uncomfort is reached. To investigate passive ROM, the footplate will be moved by the operator in very slow, isokinetic conditions from maximal dorsiflexion to maximal plantarflexion, and return (hysteresis analysis).

Muscle Architecture at rest:

The muscle architecture (fibre length and pennation angle) will be observed in vivo on the Gastrocnemius medialis (GM) and soleus (SOL) muscles at rest by using B-mode ultrasonography. Scans will be taken at 50% of the GM muscle length in the mid-sagittal plane, and probe placements will be recorded onto an acetate paper sheet to ensure the reliability of the measurements in subsequent testing session. A suitable scanning depth will be chosen sufficient to visualize both GM and SOL in the same scan and two focal zones should be used to ensure that both muscles are in focus. Two images at rest will be acquired ensuring that the muscle fibre fascicles are clearly identifiable in the scan.

M response

Central and peripheral adaptations will be examined by studying the maximal compound muscle action potential (maximal M-wave from PNS technique) and its associated twitch using a portable ergometer. The maximal M-wave will be obtained by progressive increases in intensity of single electrical stimulations (400 V, pulse width = 1 ms), evoked on relaxed muscle. Stimulation intensity will be progressively delivered from 0 to a maximum of 100mA according to the maximal soleus EMG response. Stimulations will be delivered percutaneously over the posterior tibial nerve, in the popliteal fossa, with a stainless steel ball (1-cm diameter) as monopolar cathodal electrode, connected to a constant current stimulator (Digitimer, model DS7, Welwyn Garden City, UK). The cathode (a 4 × 5 cm plate covered with electrolyte gel) will be placed on the distal part of the thigh, proximal to the patella.

Activation capacity (AC):

Maximal voluntary activation and post-activation potentiation will be assessed using the twitch interpolation technique at ankle and knee joint angles of 90° and 120° respectively. Single supra-maximal electrical pulses (pulse width = 1 ms) (i.e. an intensity 20% higher than the maximal intensity allowing getting maximal M-wave of the triceps surae) will be applied before, during and after “ramp and hold” submaximal and maximal isometric voluntary contractions (target torques: 25%, 50%, 75%, and 100% MVC). The activation capacity will then be quantified as follow:

Activation (%) = [1 - (superimposed twitch / potentiated post-contraction twitch)] × 100

Musculo- tendinous stiffness

Elastic properties of the triceps surae musculo-tendinous complex will be assessed by means of the quick-release technique adapted for in vivo experiments (Goubel and Pertuzon, 1973). Quick-release movements will be achieved by a sudden release of the footplate while the subject will maintain a submaximal voluntary isometric torque under plantarflexion. Three attempts will be performed at 30%, 60%, and 80% MVC, in a random order with two minutes rest between each trial.

Tendon and aponeurosis mechanical properties

Several studies showed that immobilization results in impaired mechanical properties (Almeida-Silveira et al., 2000, Reeves et al., 2005). However, the studies having investigated the human Achilles tendon *in vivo* (Reeves et al., 2005, Seynnes et al., 2008), placed the Ultrasound-Probe at the myotendinous junction of the gastrocnemius medialis muscle. Thus, the observed changes are attributed to changes in the free Achilles tendon and the aponeurosis. In order to evaluate the effects of immobilization on both structures separately the following study should be performed.

Stiffness is generally used to describe the mechanical properties of a tendon and is defined as the force needed to induce a certain deformation. Deformation of the free Achilles tendon and aponeurosis is measured using B-Mode Ultrasonography during an isometric contraction of the plantarflexors (Albracht & Arampatzis, 2013). In order to separate between both structures three to five successful ramp contractions are necessary with the ultrasound probe placed at the myotendinous junction of the gastrocnemius medialis muscle (Position 1) and at the myotendinous junction of the soleus muscle (Position 2). Three additional contractions are necessary to quantify the movement of the calcaneus in relation to the skin with the probe placed at the calcaneus (Position 3) (Fig. 1). The isometric contractions are performed with the knee joint 90° flexed and the ankle joint 10° dorsiflexed. The muscle force should be increased gradually within 3s up to 90% of their maximum effort. The generated plantarflexion moment is measured using a custom-made dynamometer and used to calculate tendon force.

For tendon preconditioning and warm up, 15 sub-maximum and 3 maximum voluntary (MVC) contractions are performed. The three MVCs are used to determine the maximum effort. One minute rest is given between each contraction.

During a contraction of the plantarflexors the antagonist muscles are active and might significantly influence the measured moments. Therefore, bipolar surface electrodes (EMG) are used to measure the activity of the tibialis anterior muscle during the isometric plantar flexion contractions. An estimation of the corresponding dorsiflexion moment is performed by recording the activity of the tibialis anterior during two additional sub-maximum dorsiflexons, in which the produced dorsiflexion moment shows an amplitude of the m. tibialis anterior slightly below and above the

maximal amplitude achieved during plantarflexion. Usually dorsiflexion moments of 3 to 5 and 10 to 15 Nm are suitable.

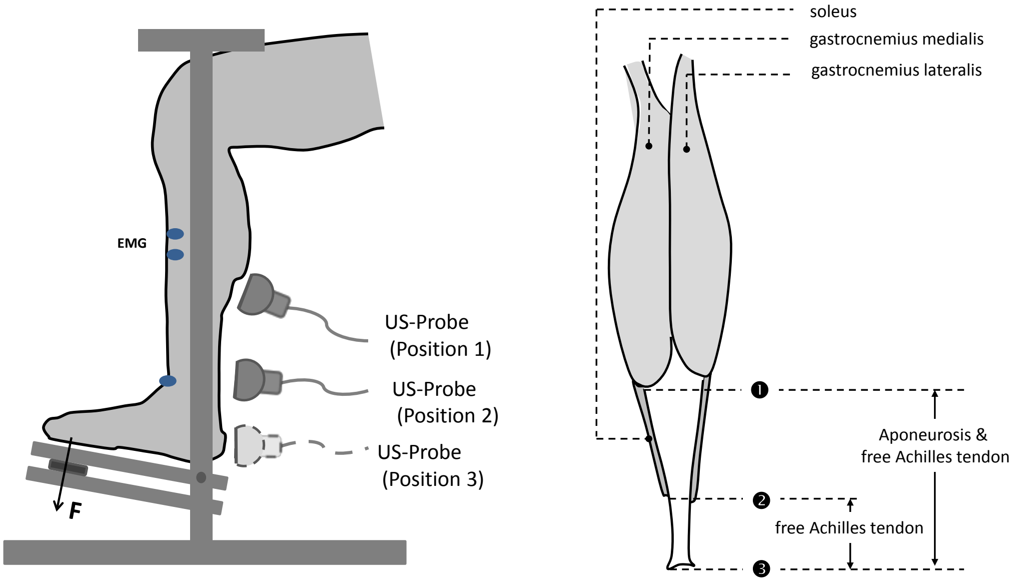

Figure 1: *The three positions of the ultrasound probe during the isometric contractions of the plantarflexors*

Fasciculations

Fasciculations are important in clinical diagnosics of motor neuron desease. Usually fascicluation have been detected by visual inspection, eloctromyography or ultrasonography. To investigate, whether immobilization effects fasciculation ultrasound clips should be recorded from the resting M. triceps suare muscles for about 3 to 4 minutes.

3 The following legislation is applicable

- Declaration of Helsinki in its latest version
- Medizinproduktegesetz (MPG)
- Medizinprodukte-Betreiber Verordnung (MPBetrV)
- Datenschutzgesetz
- Röntgenschutz-Verordnung (RöV)

4 The Intended duration of Proposed Study

The pilot study shall be performed between June and August 2013. For the main study, the intervention phase will last 60 days. Four weeks before the intervention phase the base line data are collected and follow-up examinations will be performed up to three weeks after the interventional phase. The study will take place between April 2014 and December 2014.

5 Subjects Selection

The subjects should be healthy men between 20 and 45 years of age. All subjects will be selected according the following criteria:

5.1 Inclusion Criteria for the Pre-Study

- Female and male volunteers
- Age: between 20 and 45 years
- Body mass index (BMI): 20-28 kg/m²
- Fasting blood glucose within normal ranges (70-99 mg/dl; 29-42 mmol/mol Hb)
- Glycated hemoglobin HbA1c within normal ranges (4-6%)
- Agreement and signed informed consent before the study

5.2 Exclusion Criteria for the Pre-Study

- Diabetes mellitus
- Increased bleeding tendency (hemophilia, regular use of anticoagulants)
- Allergy to nuts, legume or milk protein
- Drug, medication or alcohol abuse (frequent consumption of more than 20-30g alcohol/day)
- Intake of medication during the study
- Smoker
- Competitive athletes
- Extreme eating habits (vegan, special diets)

- Any other condition classed as unsuitable by the executive medical director

5.3 Inclusion Criteria for the Main Study

- Male volunteers
- Age: between 20 and 45 years
- Body mass index (BMI): 20 -26 kg/m^2
- Whole body fat < 25%
- Agreement and signed consent before the study

5.4 Exclusion Criteria for the Main Study

- Smoker
- Competitive Athletes
- Diabetes mellitus
- Rheumatic disease
- Muscle or joint disease
- Bone fractures in the period up to one year before study start
- Herniated disc
- Flatfeet (pes planus)
- Allergy to nuts or legume
- Vascular diseases
- Epilepsy
- Severe hyperlipidemia
- Anemia (< Hb standard values; standard values healthy men: 13.5 -17.5 g/dl) *
- Increased thrombosis risk *
- Kidney disorder: deviations from normal values for creatinine in plasma. (Normal value: Creatinine < 1.20 mg/dl)*
- Lesions of the cruciate ligaments, or orthopedic surgery in the past 10 years on the side of the Hephaistos orthosis
- Achilles tendon injury, or rupture in the past 10 years on the side of the orthosis

- hyper-/hypocalcaemia (abnormal levels of calcium in the blood, normal level: 2.15 – 2.64 mmol/l)
- Intake of anti-inflammatory drugs during the study
- Abuse of drugs or alcohol (> than 20-30g alcohol/day)
- Participation in another clinical study within the last 2 months before the start of this study
- Increased bleeding tendency (hemophilia, regular use of anticoagulants)
- History of intolerance to local anesthetics
- Imprisoned during study
- Any other condition classed as unsuitable for study participation by the medical executive director
- prior convictions (objectionable criminal record)

5.5 Additional Exclusion Criteria for MRI

- Metal implants or other osteosynthesis material
- Pacemaker
- Claustrophobia

*A thrombosis risk reduction approach will be conducted according to clinical standards: in the anamnesis, the test subject will be extensively questioned for thrombosis risk factors and assessed by means of specific analytical laboratory methods for assessing thrombosis risk using thrombophilia screening (AT III, protein C and S activities, factor V Leiden, prothrombin mutants, lupus PTT). In agreement with §5 GenDG all parameters are analyzed by a qualified lab (Labor Quade, Köln, Germany, certificate please see 10 - Appendix). In agreement with §§ 7-11 GenDG all guidelines are followed regarding clarification, subjects agreement as well as notification of the results and advisory service.

* Thrombophilia screening. This examination includes a gene diagnostic screening following the Gendiagnostikgesetz (GenDG) from 01.02.2010. The diagnostics are restricted to mutations in factor V, which will give information about blood clotting

properties. This technique is part of the routine examination. In agreement with §5 GenDG all parameters are analyzed by a qualified lab (Labor Quade, Köln, Germany, certificate please see 10 - Appendix). In agreement with §§ 7-11 GenDG all guidelines are followed regarding clarification, subjects agreement as well as notification of the results and advisory service.

5.6 Statistical Analysis

Statistical testing of the main hypotheses will be carried out with Student's t-test, comparing the changes from baseline to after the immobilization phase. Where feasible (pQCT, blood sampling and MRI scanning), baseline testing will be carried out twice in order to improve reliability of the effect estimation. Recovery of changes shall be assessed with linear mixed effect models, with time and intervention (countermeasure *vs.* control) as fixed effects and subject as random effect. All statistical analyses shall be carried out with the software 'R' (http://www.r-project.org). In the last Hephaistos study a bone loss of around 2.0% (HEP study, DLR, 2010, Ethical Application 2010169, Ärztekammer Nordrhein) with a standard deviation of 1.7% was observed. Hence, a two-sided t-test with $\alpha = 0.05$ und $\beta = 0.2$ results in a number of cases of 12 subjects per group.

5.7 Number of Subjects

The study is going to be implemented with a control and an intervention group, each consisting of 12 subjects.

5.8 Inclusion of subjects under the age of 50 (§28d Abs. 3 RöV)

One of the aims of the study is to investigate the influence of neuromuscular electrical stimulation and protein-rich lupin seeds on the loss of bone mass due to immobilization. In case of a positive effect it would be a benefit for both clinical (e.g. immobilized patients) and pre-clinical (e.g. bone loss in astronauts) applications. In addition, the expected radiation exposure is very low (<0.02mSv). This corresponds

to less than a hundredth of the yearly radiation exposure. Hence, the application is related with a very low risk and therefore scientifically and ethically justifiable.

It is necessary to include subjects under the age of 50, since the aim of the study is to investigate changes in insulin- and glucose metabolism due to the presented interventions and that great variability in insulin and glucose metabolism can be observed with increasing age.

5.9 Criteria for Randomizations

The subjects will wear the orthosis on one leg only. The side of intervention, as well as assignment to the control or countermeasure group will be done randomly with blocking. Subjects will be selected according to the criteria listed in 5.1 and 5.2.

The coding of the subjects will follow the order of arrival of the subjects on the first day of examination. Subjects who drop out of the study after inclusion will be referred to as “drop-out”. Reasons for dropping out of the ongoing study are listed in 5.6.

5.10 Drop-out Reasons:

Any of the following conditions during the study or measurements:

- Appearance of new diseases
- Any evidence that cannot justify the continuation of the study
- Emergency during the measurements
- Whenever requested by the subjects.

6 Potential Adverse Effects

6.1 Complications and risk resulting from methods

6.1.1 Neuromuscular Electrical Stimulation (NMES)

There are no known risks or side effects of the NMES procedures described above. Redness at the electrode sites is normal and should disappear within 20 min after they are removed. Also, moderate delayed-onset muscle soreness could be felt in the stimulated muscles 24-48 h after the very first NMES sessions (week 1), and is normally resolved after a further 24-48 h.

6.1.2 Lupine Seeds

Lupine belongs to the family of legume and can therefore lead to an allergic reaction. To minimize the risk of an allergic reaction allergy subjects with a known allergy to nuts or legume are excluded from the study.

6.1.3 Muscle Biopsies

In our experience (567 biopsies by Dr. Rittweger as of 2013-04-07), the greatest risks are apprehension and anxiety. The most outstanding complication have been three observers who fainted. Therefore, any new observers in this study will be instructed to use a chair. Specific efforts shall be made to generate an anxiety-relieving atmosphere in the lab. With regards to medical complications, there is a theoretical risk of infection. However, this risk is very remote (unheard of in the context of human physiology studies), and it shall be controlled by adhering to hygienic standards and working under sterile conditions. There is also a risk of bleeding and hematoma. To the best of our knowledge, there are no published data on the rate of hematoma after muscle biopsies, but it has happened only once in Dr. Rittweger’s practice that a hematoma occurred that was reported as painful. We will control this risk by screening for clotting disorders during study inclusion, by being careful with incision and tissue harvesting, and by applying a compression bandage

as stated above. Moreover, we plan to start collecting data on the relative risk of hematoma and bleeding as per this study. While doing so, we also wish to gather the data on scar size and possible areas of numbness due to peripheral skin nerve damage. Both of these risks are associated with any surgical approach to the skin, and we will control them by minimizing the incisions, and by incising along the cleavage lines. Again, there are no published data available on this topic, and we therefore plan to gather data on these effects as well.

6.1.4 Microdialysis

The microdialysis procedure is almost free of risks and complications. After implantation, reversible reddening of the skin may occur. In rare instances, local infections may occur. In own examinations such infections have, however, not happened to date. The implantation of microdialysis probes can lead to bleeding, infection and nerve damage. These complications are, however, very rare and have never happened in our 10 years of practice and experience with this technique.

6.1.5 Hyperinsulinemic Euglycemic Clamp

The hyperinsulinemic euglycemic clamp could cause low blood sugar. To avoid this, blood glucose concentration is continuously monitored and adjusted if necessary. Furthermore, the insertion of a cannula involves the risk of bruising, rarely fainting or an infection. These risks are minimized by trained personal and sterile material. After the clamp is stopped there is the risk of a potential carry-over effect of the insulin infusion. To avoid this, subjects will be served a meal to neutralize such effects.

6.1.6 Basal Metabolic Rate

The measurement of basal metabolic rate is a purely conservative, non-invasive measurement method in which only the composition of the inspired and expired air is determined. The test subjects are not exposed to any risk at all.

6.1.7 Bone densitometry

As mentioned above, the present study also involves administration of X-rays. We expect the total whole body effective dose to be below 50 μSv. This could be compared to the annual background radiation of more than 2000 μSv, or to the dose involved in air flight traveling, which is approximately 1 μSv per hour of flight. The amount of radiation inflicted by the study protocol therefore seems to be very small.

An application for using x-ray in proposed study has been submitted to 'Bundesamt für Strahlenschutz' as well.

6.1.8 Maximum Voluntary Contractions (MVC)

Maximum muscle contractions principally include the risk of damage in muscles and tendons. However, in case of voluntary contraction by healthy subjects this risk is very low. Only subjects are included who had no injuries of the lower legs, the ankles of the feet during for minimum 1 year.
Occasionally, subjects suffer from mild muscle soreness the day after maximum muscle testing.

6.1.9 Magnetic Resonance Imaging (MRI)

Magnetic resonance techniques generally use a constant magnetic field (3 Tesla), magnetic field gradients, and RF-radiation. The physical interaction with the human body is limited by the hardware to avoid e.g. overheating, and is fully reversed immediately after the experiment.
Special risks are caused by the attraction of ferromagnetic materials and the development of heat by electrical eddy currents in conducting e.g. metallic materials. Therefore subjects with ferromagnetic metallic or electronic materials are not included into the study. Before an examination subjects must take off all metallic parts of clothing, jewellery, and ornaments. The entrance of the MR-cabin is equipped with a metal-detector.

6.1.10 Vertical Jumping Test

As with any sports or physical activity, there is a very remote possibility of injury, i.e. muscle tear or strains or sprains or the ankle or other joints. This risk will be controlled by appropriate instruction and supervision of test subjects, as well as by wearing appropriate footwear.

6.1.11 Blood

The blood sampling carries with it the usual risks, such as bruising or venous inflammation at the site of puncture by the catheter or injury. Vasovagal syncope could occur, and members of staff have received training to deal with this condition, which in itself is not dangerous.

6.1.12 The Hephaistos Orhtosis

During the 60 days of intervention, muscle loss is expected to occur on the side of intervention. However, this is not dangerous in itself and expected to fully recover. To keep the risk as low as possible, subjects will be assisted by a physiotherapist when they take off the orthosis at the end of the intervention time. They will perform their first steps together with the physiotherapist and will be trained on some exercises to build up the muscle. In the last study only one of the subjects showed nerval problems which turned out to be due to flat feet (pes planus). Therefore, subjects with flat feet will be precluded from this study (see list of exclusion criteria). None of the other subjects showed any problems so that the risk for healthy subjects recruited according to the exclusion criteria listed in 5.2 are very low.

6.1.13 Ergometric device / Quick release

The specific portable ergometer has already been presented, and validated in a scientific publication (Lambertz et al., 2008), and has already been used to assess changes in neuromechanical properties in clinical studies performed between 2007 and 2012 and approved by the ethic committee of the Picardy region (France) (N°

Afssaps: 2007-A00186-47, N° protocol code: 110892). The identified risks of using this portable ergometer are:

Biological/mechanical risks: under static contraction, as there is no joint movement, the only risk would be these identified in 6.1.8 during maximal voluntary contractions. Under dynamic movement (induced by quick-release technique), the movement can be surprising and uncomfortable for the subject (as the foot plate will be released suddenly while the subject will maintain a sub-maximal target torque) but we have never had any incident since 20 years of using such method in different populations (children, elderly, healthy, pathological, …) as mechanical end stop are positioned according to the subject’s ankle range of motion before conducting quick-release test.

Electrical risks: on the whole ergometric device the only parts electrically connected are the tension-compression load cell (TEDEA 614 "S" Type) (12v), and the 8.5w holding magnet (Mécalectro 58201) (24v).

6.1.14 Passive Range of Motion

The movement amplitude being manually and slowly performed by the operator, and the amplitude excursion ending on subject instruction when reaching uncomfort due to musculo-tendinous stretching tension, there are no any perceived risk in performing this test.

6.1.15 Muscle Architecture at Rest

A standard medical ultrasound device will be adopted in this study (As in 6.1.14). There are no know risks or side effects of using ultrasound probe with electrolytic gel in healthy subjects.

6.1.16 Peripheral Nerve Stimulation (PNS)

The device used to induce electrical stimulation is a medical isolated stimulator. Thus there are no risks for the subject to be in contact with 220v. The intensity will be progressively increased. The risks for the subjects are an uncomfortable feeling at

the stimulation electrode position. As for NMES (6.1.1) the same inconvenient should be identified following PNS.

6.1.17 Activation Capacity (AC)

Except the identified risks reported for maximal voluntary contraction (6.1.8), the subject can perceive the supra-maximal stimulation of the posterior tibial nerve at rest and during voluntary contractions as uncomfortable. However, this protocol has already been applied on children (7-10 years-old) without any adverse effect and compliance.

6.1.18 Transkranielle Magnetstimulation

There are no known risks for healthy subjects recruited according to the exclusion criteria listed in 5.2.

6.1.19 Tendon and Aponeurosis Mechanical Properties

Ultrasonography

A standard medical ultrasound device will be adopted in this study. Clinically, ultrasound diagnostics are being widely used – a risk-free, painless and non-invasive imaging technique. We cannot see any complications or risks arising from this procedure.

Isometric plantarflexion contraction

Theoretically, the risk of muscle and tendon injuries exists during the isometric ramp contractions. However, this risk is deemed to be very low. In case this very rare situation occurs, the first aid is always prepared.

7 Risk-Benefit Analysis

We have detailed the risks that might theoretically occur as a consequence of this study in section 6. Most of the risks are either negligible or not substantial. Moreover,

the researchers and the staff involved in this study are highly qualified and well experienced in order to cope with these risks. Reasonable efforts are being made in order to mitigate and reduce the risks amongst others by the exclusion criteria listed in 5.2.

Impairment of the glucose metabolism and the development of insulin resistance is a severe problem in many people, in particular in those who undergo immobilization such as bed rest or space flight or even as a consequence of physical injury. This study shall help to get further insights into the occurring mechanisms which lead to insulin resistance and to develop new therapies. In the past it could be shown that both, lupine seeds and neuromuscular electrical stimulation on their own do have a positive effect on the blood glucose levels. Whether this principle mechanism is also effective in immobilization remains to be determined. While the mechanism of the lupine seeds is still not clear, evidence suggests that neuromuscular electrical stimulation improves the glucose uptake in the muscle. By combining both stimuli the glucose lowering effect shall be enhanced and analyzing the GLUT4 recruiting pathways shall help to learn more about the actual mechanisms. Findings resulting from this study will help to improve the prevention of the development of insulin resistance and could even be interesting for the therapy of existing insulin resistance such as in diabetes type 2. Of note, secondary detrimental effects of diabetes have become a major burden to society.

It should also be mentioned here that we can reach a local immobilization with the Hephaistos orthosis, while the rest of the body is not impaired. In consequence, the health, psychological and social risk of the presented study can be rated less than in bed rest studies, which are normally implemented to simulate immobilization. Thus, it seems very justifiable to state that the risks involved in this study are small in comparison to the benefits.

8 Insurance Protection

The presented study is an epidemiological study which makes less demands on the insurance company than clinical studies following §40 AMG or §20 MPG.

Nevertheless, subjects are insured during the study by an insured sum of 5 million Euros.

a) Berufsgenossenschaftliche Versicherung für Unfälle auf dem Gelände des DLR in Zusammenhang mit der Tätigkeit als Versuchperson:
Berufsgenossenschaft Energie Textil Elektro Medienerzeugnisse (BG ETEM)
Gustav-Heinemann-Ufer 130
50968 Köln
Tel.: 0221-3778; Fax.: 0221- 342503
Nummer 726.419-4

b) Betriebs-Haftpflichtversicherung:
HDI Gerling Industrie Versicherung AG
Am Schönenkamp 45,
40599 Düsseldorf,
Tel.: 0221-7482-5404, Fax: 0511-645-1150023
Versicherungsnummer: 39 904419 03394 390

c) Allgemeine Unfallversicherung und Wegeversicherung:
Allianz Versicherungs-AG
Ludwigstr. 21
80539 München
Tel.: 089- 2302-0; Fax.: 089- 23022615
Nummer PU 70/0501/3713200/502

9 Signature

The undersigned agree to the protocol in the mentioned conditions for carrying out the study

Director of the
Institute of Aerospace Medicine:

Prof. Dr. med. Rupert Gerzer

Applicant and executive medical director
(Ärztlicher Leiter der Prüfung):

Prof. Dr. Jörn Rittweger

Scientists:

Prof. Dr. Jörn Rittweger

Dr. Petra Frings-Meuthen

PD Dr. Jochen Zange

Dr. Bergita Ganse

Darius Kornetka

Kathrin Schopen

Ann Charlotte Ewald

Prof. Dr. med. Wilhelm Bloch

Dr. Nicola Maffiuletti

Dr. Michael Boschmann

Dr. Christian Leukel

Jun.-Prof. Dr. Kirsten Albracht

10 Appendix

(1) A letter to the Ethics Commission

(2) Statement for RöV

(3) Subjects information for the Pre-Study

(4) Subject information for the Main Study

(5) Insurance

(6) Evidence of two years of experience in the application of X-rays

(7) Evidence of the requisite qualification in radiation protection

(8) List of investigators

(9) CVs of investigators

(10) Subjects Consent for the Pre-Study

(11) Subjects Consent for the Main-Study

(12) Subjects contract for the Main Study

(13) Privacy Statement

(14) Consent for Vision and Sound Material for the Pre-Study

(15) Consent for Vision and Sound Material for the Main-Study

(16) Thrombophilia Screening

(17) Consent to the screening of blood for HIV and hepatitis for the Pre-Study

(18) Consent to the screening of blood for HIV and hepatitis for the Main-Study

(19) Confidentially undertaking DLR for the Pre-Study

(20) Confidentially undertaking DLR for the Main-Study

(21) Indication of orthosis handling

(22) Declaration of conformity for digitimer limited

(23) Declaration of conformity by ORTEMA

(24) MEDCert Certificate for DLR

11 References

www.mdialysis.com

Alibegovic AC, Højbjerre L, Sonne MP, van Hall G, Stallknecht B, Dela F, Vaag A. (2009) Impact of 9 days of bed rest on hepatic and peripheral insulin action, insulin secretion, and whole-body lipolysis in healthy young male offspring of patients with type 2 diabetes. Diabetes; 58(12):2749-56

Almeida-Silveira MI, Lambertz D, Perot C, and Goubel F. (2000), Changes in stiffness induced by hindlimb suspension in rat Achilles tendon. *Eur J Appl Physiol* 81: 252–257.

Bertoglio JC, Calvo MA, Hancke JL, Burgos RA, Riva A, Morazzoni P, Ponzone C, Magni C, Duranti M. (2011) Hypoglycemic effect of lupin seed γ-conglutin in experimental animals and healthy human subjects. Fitoterapia.; 82(7):933-8.

Bindman, L.J., Lippold, O.C. & Redfearn, J.W. (1964) The Action of Brief Polarizing Currents on the Cerebral Cortex of the Rat (1) during Current Flow and (2) in the Production of Long-Lasting after-Effects. *J Physiol*, **172**, 369-382.

Blanc S, Normand S, Pachiaudi C, Fortrat JO, Laville M, Gharib C. (2000) Fuel homeostasis during physical inactivity induced by bed rest. J Clin Endocrinol Metab.; 85(6):2223-33.

Botter A, Oprandi G, Lanfranco F, Allasia S, Maffiuletti NA, Minetto MA. Atlas of the muscle motor points for the lower limb: implications for electrical stimulation procedures and electrode positioning. Eur J Appl Physiol. 2011;111(10):2461-71.

Elbert, T., Lutzenberger, W., Rockstroh, B. & Birbaumer, N. (1981) The influence of low-level transcortical DC-currents on response speed in humans. *Int J Neurosci*, **14**, 101-114.

Frid AH, Nilsson M, Holst JJ, Björck IM. (2005) Effect of whey on blood glucose and insulin responses to composite breakfast and lunch meals in type 2 diabetic subjects. Am J Clin Nutr.; 82(1):69-75.

Goubel F, and Pertuzon E (1973). [Evaluation of the elasticity of muscle in situ by the quick-release method]. Arch Int Physiol Biochim 81: 697-707.

Lambertz D, Paiva MG, Marinho SM, Aragão RS, Barros KM, Manhães-de-Castro R, Khider N, Canon F (2008). A reproducibility study on musculotendinous stiffness quantification, using a new transportable ankle ergometer device. J Biomech. 14;41(15):3270-3.

Lovati MR, Manzoni C, Castiglioni S, Parolari A, Magni C, Duranti M. (2011) Lupin seed γ-conglutin lowers blood glucose in hyperglycaemic rats and increases glucose consumption of HepG2 cells. Br J Nutr.; 107(1):67-73.

Nitsche, M.A., Fricke, K., Henschke, U., Schlitterlau, A., Liebetanz, D., Lang, N., Henning, S., Tergau, F. & Paulus, W. (2003) Pharmacological modulation of cortical excitability shifts induced by transcranial direct current stimulation in humans. *J Physiol*, **553**, 293-301.

Nitsche, M.A. & Paulus, W. (2000) Excitability changes induced in the human motor cortex by weak transcranial direct current stimulation. *J Physiol*, **527 Pt 3**, 633-639.

Reeves ND, Maganaris CN, Ferretti G, Narici MV(2005), Influence of 90-day simulated microgravity on human tendon mechanical properties and the effect of resistive countermeasures. J Appl Physiol., 98(6):2278-86

Reis, J., Schambra, H.M., Cohen, L.G., Buch, E.R., Fritsch, B., Zarahn, E., Celnik, P.A. & Krakauer, J.W. (2009) Noninvasive cortical stimulation enhances motor skill acquisition over multiple days through an effect on consolidation. *Proc Natl Acad Sci U S A*, **106**, 1590-1595.

Rothwell, J.C., Day, B.L., Thompson, P.D. & Kujirai, T. (2009) Short latency intracortical inhibition: one of the most popular tools in human motor neurophysiology. *J Physiol*, **587**, 11-12.

Purpura, D.P. & McMurtry, J.G. (1965) Intracellular Activities and Evoked Potential Changes during Polarization of Motor Cortex. *J Neurophysiol*, **28**, 166-185.

Seynnes, OR, Maffiuletti NA, Maganaris CN, de Boer MD, Pensini M, di Prampero PE, Narici MV (2008). Soleus T reflex modulation in response to spinal and tendinous adaptations to unilateral lower limb suspension in humans. Acta Physiol, 194(3):239-51.

Stagg, C.J. & Nitsche, M.A. (2011) Physiological basis of transcranial direct current stimulation. The Neuroscientist : a review journal bringing neurobiology, neurology and psychiatry, **17**, 37-53.

Stein TP, Schulter MD, Boden G. (1994) Development of insulin resistance by astronauts during spaceflight. Aviat Space Environ Med.; 65(12):1091-6.

Stein TP, Schluter MD. (1997) Human skeletal muscle protein breakdown during spaceflight. Am J Physiol.; 272(4 Pt 1):E688-95.

Weber-Carstens S, Schneider J, Wollersheim T, Assmann A, Bierbrauer J, Marg A, Al Hasani H, Chadt A, Wenzel K, Koch S, Fielitz J, Kleber C, Faust K, Mai K, Spies CD, Luft FC, Boschmann M, Spranger J, Spuler S. (2013) Critical illness myopathy and GLUT4: significance of insulin and muscle contraction. Am J Respir Crit Care Med.; 187(4):387-96.
